# On boundary damping to reduce the rain–wind oscillations of an inclined cable with small bending stiffness

**DOI:** 10.1007/s11071-018-4596-0

**Published:** 2018-10-24

**Authors:** Tugce Akkaya, Wim T. van Horssen

**Affiliations:** 1 Department of Mathematical Physics, Delft Institute of Applied Mathematics, Delft University of Technology, Delft, The Netherlands; 2 Present Address: Department of Applied Mathematics, University of Twente, Enschede, The Netherlands

**Keywords:** Tensioned Euler–Bernoulli beam, Time-varying mass, Rain–wind-induced vibrations of an inclined cable

## Abstract

In this paper, a model will be derived to describe the rain–wind-induced oscillations of an inclined cable. Water rivulets running along the cable and aerodynamics forces acting on the cable are taken into account to describe these oscillations. A boundary damper is assumed to be present near the lower endpoint of the cable. For a linearly formulated initial-boundary value problem for a tensioned beam equation describing the in-plane transversal oscillations of the cable, the effectiveness of this damper is determined by using a two-timescales perturbation method. It is shown how mode interactions play an important role in the dynamic behaviour of the cable system. Some resonant and non-resonant cases have been studied in detail.

## Introduction

The study on how to damp vibrations in stay cables, which are attached to a pylon at one end and to a bridge deck at the other end, is of great importance not only in structural engineering but also in applied mathematics. The combined effect of rain and of wind can change the aerodynamic properties of the cable-stayed bridge and can lead to relatively large amplitude vibrations of the cables. For example, one can refer to the Erasmus bridge in Rotterdam, which started to vibrate heavily under mild wind–rain conditions shortly after its opening in 1996. This bridge was temporarily closed to the traffic as a safety precaution. As a temporarily measure, polypropylene ropes were installed between the cables and the bridge desk. Later, these ropes were replaced by hydraulic dampers as a permanent measure and by conforming to the aesthetic of the bridge designed by the architects. Much research on this problem has been done both numerically and experimentally to understand the mechanisms of rain–wind-induced vibration of inclined stay cables [10, 11]. For more recent experiments and numerical computations, the reader is referred to the following articles [5, 8, 13, 18].

**Fig. 1:**
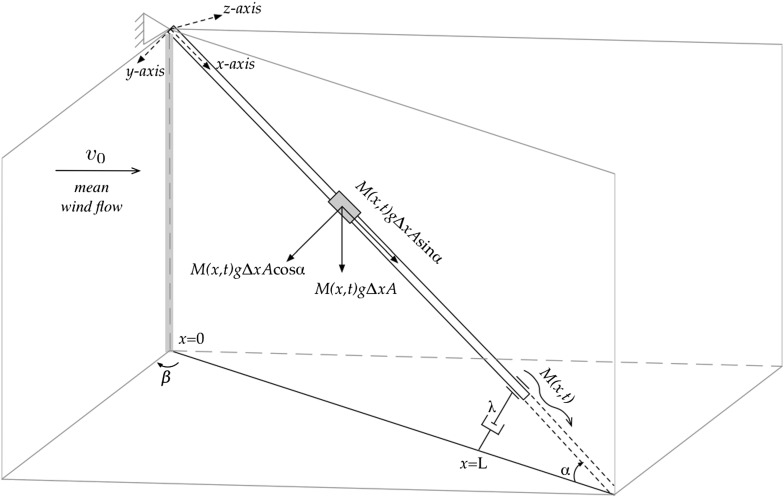
The inclined stretched cable in a static state

As has been observed from wind-tunnel experiments, raindrops hitting the inclined stay cable cause the generation of one or more rivulets on the surface of the cable. The presence of flowing water on the cable changes the mass and the aerodynamic properties of the bridge system that can lead to instabilities. These water rivulets on the cable surface can be considered as a time-varying mass in the system [1, 3]. In order to suppress undesirable vibrations in bridge structures, different kinds of dampers such as tuned mass dampers and oil dampers can be installed between the cables and the bridge deck. Numerous work has been done from the theoretical and the experimental point of view to predict the optimal damper location and type. Jacquot [15] calculated the optimal value and location of the viscous damper which is located in a randomly forced horizontal cantilever beam.

These physical problems of rain–wind-induced vibration of inclined cables can be modelled mathematically by initial-boundary value problems for string-like or beam-like problems. For string-like problems, the static state due to gravity and the dynamic state due to a parametrical and a transversal excitation at one of the ends of the inclined string were studied in [7]. For the inclined cable subjected to wind with a moving rivulet on its surface, the nonlinear dynamic model is investigated by considering the equilibrium position of the rivulet [19]. The effect of the static condensation of the longitudinal displacement due to the cable inclination and the cable total tension is investigated by using numerical and analytical techniques in [25]. In addition, the interaction among the three excitation sources: self excitation, which is caused by a mean wind flow, and external and parametric excitations due to vertical motion of the ground support, on the nonlinear dynamics of the inclined cable related to a cable-stayed bridge were studied in [20]. In many papers, the rain–wind-induced vibrations of inclined taut cables have been studied, but much less work has been done on the rain–wind-induced vibrations of an inclined cable subjected to wind with time-varying rivulets on its surface.

From the practical point of view, the viscoelastic materials are modelled with a combination of spring-like and dashpot-like elements at the boundary in order to represent restorative force component and damping component, respectively. In this paper, we consider the linear (and nonlinear) dynamic response due to only a damper near one of the support ends of the inclined cable. On the cable, a time-varying (rain) mass rivulet is assumed to be present (see also Fig. 1). The stationary wind flow will lead to the phenomenon of self excitation of the cable. The damper location in the cable-stayed bridge is quite close to the anchorage of the cable. For more information on the effect of the bending stiffness of a tensioned cable for varying damper locations, the reader is referred to [14, 21, 22].

The aim of this paper is to provide an understanding of how effective boundary damping is for inclined stretched beams with a small bending rigidity. These problems for strings or beams are considered to be basic models for oscillations of cables from a practical viewpoint. The outline of this paper is as follows. In Sect. 2, we apply a variational method in order to derive the governing equations of motion of the tensioned Euler–Bernoulli beam, and obtain a system of three coupled PDEs. By using Kirchhoff’s approach, the number of PDEs in the system is reduced to two PDEs (one for the in-plane motion, and one for out-of-plane motion) or to a single PDE (only for the in-plane motion). The main aim of this section is to give a model to describe the dynamics of rain–wind-induced vibrations of an inclined beam, where the gravity effect is schematically shown in Fig. 1. In Sect. 3, we only consider the in-plane motion of the inclined beam with only one rivulet on the cable. In Sect. 4, the two-timescales perturbation method is used to solve the problem and some (non)resonance frequencies are determined. In this paper, we will consider the pure resonance case and the non-resonance case. All other resonance cases can be investigated similarly. Finally, the conclusions are presented in Sect. 5.

## Equations of motion

We consider an inclined, perfectly flexible, elastic beam with a small bending rigidity on a finite interval $x\in [0,L]$, which is attached to a dashpot $\lambda$ at $x=L$, and assumed to be simply supported at $x=0$ (see Fig. 1). *u*, *w* and *v* are the displacements in *x*-direction, *y*-direction and *z*-direction, respectively. From Newton’s second law, the equations of motion can be stated as follows: the time derivative of the linear momentum of the system is equal to the sum of external forces which are elastic forces, gravity, drag and lift forces due to the uniform wind flow $v_{0}$, blowing under a yaw angle $\beta$ as can be seen in Fig. 1. It is assumed that the water rivulet is not blown off the cable. Furthermore, we assume that the tension due to stretching in the beam is large enough, such that the small sag of the beam, that is, the displacements in *x* and *z*-directions due to gravity, can be neglected. Similarly, due to this tension, we neglect the wind force along the cable in *x*-direction. Hence, the total tension in the beam at $x=x_{0}$ can be written by:

1 $$\begin{aligned} T(x_{0},t)=T_{0}+\int _{\bar{x}=x_{0}}^{L} M(\bar{x},t) g A \mathrm {sin}(\alpha )\mathrm {d}\bar{x}, \end{aligned}$$

where $T_{0}$ is the pretension in the beam, *g* is the acceleration due to gravity, *A* is the cross-sectional area of the beam, $\alpha$ is the angle between the beam and the horizontal plane, and $M(\bar{x},t))$ is the mass density of the beam including the time-varying water rivulet.

Let the coordinates (*x*, *y*, *z*) of a material point of the unstretched beam be (*x*, 0, 0) with $x\in [0,L]$, where the *x*-axis, *y*-axis and *z*-axis are defined in Fig. 1. The dynamic displacement of this material point is denoted by $u(x,t)\mathbf i$, $v(x,t)\mathbf k$ and $w(x,t)\mathbf j$, where $\mathbf i$, $\mathbf k$ and $\mathbf j$ are the unit vectors along the *x*-axis, *y*-axis and *z*-axis [7]. The vector position *R*(*x*, *t*) of this material point in the dynamic state is obtained by:

2 $$\begin{aligned} R(x,t)= & {} \left[ x+{1\over {AE}}\int _{0}^{x} [T_{0} +\int _{\bar{x}=s}^{L} M(\bar{x},t) g A \mathrm {sin}(\alpha )\mathrm {d}\bar{x}]\mathrm {d}s \right. \nonumber \\&\quad \left. +u(x,t)\right] \mathbf i +v(x,t)\mathbf j +w(x,t)\mathbf k , \end{aligned}$$

where *E* is Young’s modulus. The relative strain per unit length of the stretched beam is written by:

3 $$\begin{aligned}&\epsilon _{xo}(x,t)= \left| \frac{\partial }{\partial x}R(x,t) \right| -1,\\ \nonumber&= \sqrt{\left[ 1{+}{T_{0} \over {AE}}+{1\over {AE}}\int _{\bar{x}=x}^{L} M(\bar{x},t) g A \mathrm {sin}(\alpha )\mathrm {d}\bar{x}+u_{x}\right] ^2+v_{x}^2+w_{x}^2}-1, \end{aligned}$$

where $u_{x}$, $v_{x}$ ,and $w_{x}$ represent the derivative with respect to *x* of *u*(*x*, *t*), *v*(*x*, *t*) ,and *w*(*x*, *t*), respectively. By assuming that $u_{x}^2$ is small with respect to $u_{x}$ and $v_{x}^2+w_{x}^2$, and by expanding the square-root in a Taylor series, we have approximately

4 $$\begin{aligned} \epsilon _{xo}\approx & {} {T_{0} \over {AE}}+ {1\over {AE}}\int _{\bar{x}=x}^{L} M(\bar{x},t) g A \mathrm {sin}(\alpha )\mathrm {d}\bar{x}\nonumber \\&\quad +\, u_{x}+ {v_{x}^2 \over {2}}+\,{w_{x}^2 \over {2}}. \end{aligned}$$

For the curvature of the beam in the (*x*, *y*, *z*)-space (out of plane) or for the curvature of the beam in the (*x*, *y*)-plane (in plane), the reader is referred to [6, 23]. The axial strain of a generic point of the beam located at distances *y* and *z* (in the *y* and *z* directions) from the centre line of the beam is defined by:

5 $$\begin{aligned} {\epsilon _{xx}}\approx -y v_{xx} -z w_{xx} . \end{aligned}$$

Hence, the total strain of a line-element of the beam is approximately given by:

6 $$\begin{aligned} \epsilon _{x}&= \epsilon _{x0}+\epsilon _{xx}, \nonumber \\&\approx {T_{0} \over {AE}}+{1\over {AE}}\int _{\bar{x}=x}^{L} M(\bar{x},t) g A \mathrm {sin}(\alpha )\mathrm {d}\bar{x}\nonumber \\&\quad + u_{x}+ {v_{x}^2 \over {2}}+{w_{x}^2 \over {2}}-y v_{xx} - z w_{xx}. \end{aligned}$$

The equations of motion describing the vibrations of the beam will be obtained by using Hamilton’s principle [12]. In order to apply this principle, the kinetic energy and potential energy for the beam should be defined and calculated. The potential energy of the beam is given approximately by (using Hooke’s Law [16]) and defined as:

7 $$\begin{aligned} E_{P}&={E A \over 2} \int _{0}^{L} \Biggl [ {T_{0} \over {AE}}+ {1\over {AE}}\int _{\bar{x}=x}^{L} M(\bar{x},t) g A \mathrm {sin}(\alpha )\mathrm {d}\bar{x}\nonumber \\&\quad + u_{x}+ {v_{x}^2 \over {2}}+{w_{x}^2 \over {2}} \Biggl ]^2 \mathrm {d}x \nonumber \\&\quad +{E\over 2} \int _{0}^{L} (I_{y} v_{xx}^2 + I_{z} w_{xx}^2 )\mathrm {d}x \nonumber \\&\quad -Ag \int _{0}^{L} M(x,t) [u \mathrm {sin}(\alpha )+v \mathrm {cos}(\alpha )] \mathrm {d}x. \end{aligned}$$

where $I_{y}$ and $I_{z}$ represent the axial moments of area about the y- and z-axes, respectively. In addition, the kinetic energy of the beam is given by:

8 $$\begin{aligned} E_{K}={A \over 2}~\int _{0}^{L} M(x,t)[u_{t}^{2}+v_{t}^{2}+w_{t}^{2}] \mathrm {d}x. \end{aligned}$$

The Hamiltonian integral is $\mathcal {F}=\mathcal {F}(t_{2})-\mathcal {F}(t_{1})=\int _{t=t_{1}}^{t_{2}} (E_{K}-E_{P})\mathrm {d}t$. If we denote the integrand with $f(u,u_{x},u_{t},v,v_{x},v_{xx},v_{t},w_{x},w_{xx},w_{t})$, then the three Euler-Lagrange equations of the variational problem $\delta \mathcal {F}=0$ , which we have to solve according to the Hamiltonian principle, are as follows:

$$\begin{aligned}&\frac{\partial }{\partial x} \Big ( \frac{\partial f }{\partial u_{x}} \Big )+ \frac{\partial }{\partial t} \Big ( \frac{\partial f }{\partial u_{t}} \Big )- \frac{\partial f}{\partial u}=0,\\&\frac{\partial }{\partial x} \Big ( \frac{\partial f }{\partial v_{x}} \Big )- \frac{\partial ^2 }{\partial x^2} \Big ( \frac{\partial f }{\partial v_{xx}} \Big )+ \frac{\partial }{\partial t} \Big ( \frac{\partial f }{\partial v_{t}} \Big )-\frac{\partial f}{\partial v}=F_{y}, \\&\frac{\partial }{\partial x} \Big ( \frac{\partial f }{\partial w_{x}} \Big )- \frac{\partial ^2 }{\partial x^2} \Big ( \frac{\partial f }{\partial w_{xx}} \Big )+ \frac{\partial }{\partial t} \Big ( \frac{\partial f }{\partial w_{t}} \Big )=F_{z}, \end{aligned}$$

or equivalently, the equations of motion are given by

9 $$\begin{aligned}&\frac{\partial }{\partial t} \Big [ M(x,t) u_{t} \Big ]-E\frac{\partial }{\partial x} \Bigg [ {T_{0} \over {AE}}\nonumber \\&\quad +\frac{ 1}{E}\int _{\bar{x}=x}^{L} M(\bar{x},t) g \mathrm {sin}(\alpha )\mathrm {d}\bar{x} + u_{x}+ {v_{x}^2 \over {2}}+{w_{x}^2 \over {2}} \Bigg ] \nonumber \\&\quad -M(x,t) g \mathrm {sin}(\alpha )=0, \end{aligned}$$

10 $$\begin{aligned}&\frac{\partial }{\partial t} \Big [ M(x,t) v_{t} \Big ]-E\frac{\partial }{\partial x} \Bigg [ v_{x}\Bigg ({T_{0} \over {AE}}\nonumber \\&\quad + \frac{ 1}{E}\int _{\bar{x}=x}^{L} M(\bar{x},t) g \mathrm {sin}(\alpha )\mathrm {d}\bar{x} + u_{x}+ {v_{x}^2 \over {2}}+{w_{x}^2 \over {2}} \Bigg ) \Bigg ]\nonumber \\&\quad + \frac{\partial ^2 }{\partial x^2} \Bigg ( { E I_{y} \over A } v_{xx} \Bigg )-M(x,t)g \cos (\alpha )= \frac{F_{y}}{A}, \end{aligned}$$

11 $$\begin{aligned}&\frac{\partial }{\partial t} \Big [ M(x,t) w_{t} \Big ]-E\frac{\partial }{\partial x} \Bigg [ w_{x}\Bigg ({T_{0} \over {AE}}\nonumber \\&\quad + \frac{ 1}{E}\int _{\bar{x}=x}^{L} M(\bar{x},t) g \mathrm {sin}(\alpha )\mathrm {d}\bar{x} + u_{x}+ {v_{x}^2 \over {2}}+{w_{x}^2 \over {2}}\Bigg ) \Bigg ]\nonumber \\&\quad + \frac{\partial ^2 }{\partial x^2} \Bigg ( { E I_{z} \over A } w_{xx} \Bigg )=\frac{F_{z}}{A}, \end{aligned}$$

where $F_{y}$ and $F_{z}$ are the aerodynamic forces in the in-plane and in the out-of-plane, respectively, and are defined by

12 $$\begin{aligned} F_{y}&=-[\mathcal {D}~\mathrm {sin}(\phi )+\mathcal {L}~\mathrm {cos}(\phi )], \end{aligned}$$

13 $$\begin{aligned} F_{z}&=[\mathcal {D}~\mathrm {cos}(\phi )+\mathcal {L}~\mathrm {sin}(\phi )]. \end{aligned}$$

**Fig. 2:**
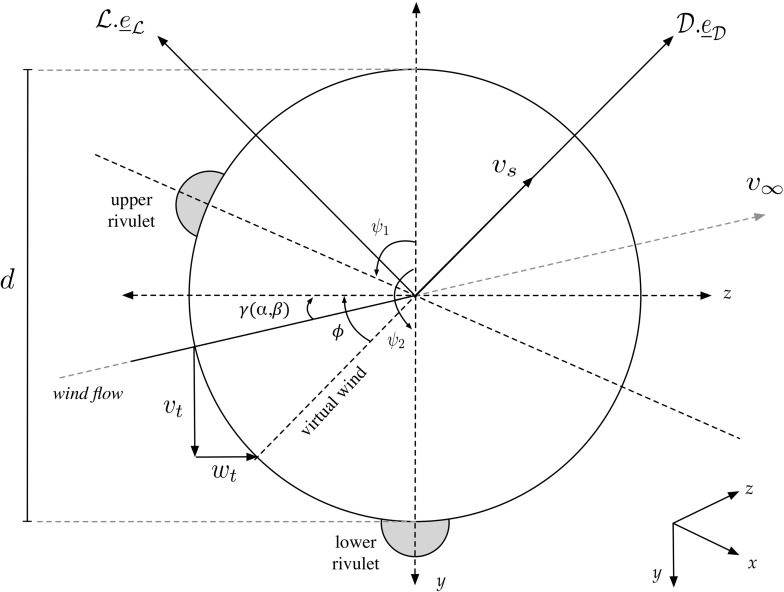
The model of the cross section of cable with rivulets

Here $\mathcal {D}$ and $\mathcal {L}$ are the magnitudes of the drag and lift forces, respectively, which may be given by:

14 $$\begin{aligned} \mathcal {D}&=\frac{1}{2} {\rho }_{a} d L c_{\mathcal {D}}(x,t;\phi _{i}^{*}) v_{s}^{2}, \end{aligned}$$

15 $$\begin{aligned} \mathcal {L}&=\frac{1}{2} {\rho }_{a} d L c_{\mathcal {L}}(x,t;\phi _{i}^{*}) v_{s}^{2}, \end{aligned}$$

where ${\rho }_{a}$ is the air density, *d* is the diameter of the cross section of the circular part of the beam cable, *L* is the length of beam, $v_{s}$ is the virtual wind velocity which is given by

16 $$\begin{aligned} v_{s}^2=(v_{\infty }\mathrm {cos}(\gamma )-w_{t})^2+(v_{\infty }\mathrm {sin}(\gamma )+v_{t})^2. \end{aligned}$$

Figure 2 shows the centre of a cross section of the cable with rivulets. $\psi _{1}$ and $\psi _{2}$ are the displacements of the upper and the lower rivulet, respectively, and $\phi _{i}^{*}$ is the wind attack angle, which is defined by $\phi _{i}^{*}=\phi -\psi _{i}$, $i=1,2$. The angle between the virtual wind velocity $v_{s}$ and the horizontal axis is defined by $\phi$, which can be expressed as:

17 $$\begin{aligned} \phi (t)&=\mathrm {arctan}\Big ( \frac{v_{\infty }\mathrm {sin}(\gamma )+v_{t}}{v_{\infty }\mathrm {cos}(\gamma )-w_{t} } \Big ),\nonumber \\&\approx \mathrm {arctan(\mathrm {tan}(\gamma ))}+ \frac{v_{t}}{v_{\infty }} \mathrm {cos}(\gamma )+ \frac{w_{t}}{v_{\infty }} \mathrm {sin}(\gamma )\nonumber \\&\quad -\frac{v_{t}^2}{v_{\infty }^2} \mathrm {sin}(\gamma )\mathrm {cos}(\gamma ) +\frac{v_{t}w_{t}}{v_{\infty }^2}\Big ( 2 \mathrm {cos}^2(\gamma )-1 \Big ) \nonumber \\&\quad +\frac{w_{t}^2}{v_{\infty }^2} \mathrm {sin}(\gamma )\mathrm {cos}(\gamma ) + \frac{v_{t}^3}{v_{\infty }^3} \Big ( \mathrm {cos}(\gamma )-\frac{4}{3} \mathrm {cos}^3(\gamma ) \Big )\nonumber \\&\quad + \frac{v_{t}^2 w_{t}}{v_{\infty }^3}\Big (\mathrm {sin}(\gamma )-4\mathrm {cos}^2(\gamma )\mathrm {sin}(\gamma ) \Big ) \nonumber \\&\quad + \frac{v_{t} w_{t}^2}{v_{\infty }^3}\Big (4\mathrm {cos}^3(\gamma )-3\mathrm {cos}(\gamma ) \Big ) \nonumber \\&\quad + \frac{w_{t}^3}{v_{\infty }^3}\Big (\frac{4}{3} \mathrm {cos}^2(\gamma )\mathrm {sin}(\gamma )-\frac{1}{3} \mathrm {sin}(\gamma )\Big )+\cdots ,\nonumber \\ \end{aligned}$$

where $v_{\infty }= v_{0}\sqrt{\mathrm {cos}^2(\beta )+\mathrm {sin}^2(\alpha )\mathrm {sin}^2(\beta )}$ is the effective wind flow, and $\gamma$ is the angle of attack, which depends on the inclination angle $\alpha$ and the yaw angle $\beta$ defined by Geurts and van Staalduinen [11]

18 $$\begin{aligned} \gamma (\alpha ,\beta )= \mathrm {arcsin} \Big ( \frac{\mathrm {sin}(\alpha )\mathrm {sin}(\beta )}{\sqrt{\mathrm {cos}^2(\beta )+\mathrm {sin}^2(\alpha )\mathrm {sin}^2(\beta )}} \Big ). \end{aligned}$$

As can be seen in Fig. 2, the position of the upper and lower rivulet is determined by $\psi _{1}$ and $\psi _{2}$, respectively. From experimental data [4], we may assume that the total time-varying mass due to these rivulets on the cable changes periodically and may be defined to have the form [1, 2]

19 $$\begin{aligned} m_{1}(x,t)&=M_{1}\mu _{1}(x,t)\nonumber \\&=M_{1}~(1+A_{1}~\mathrm {sin}(\gamma _{1}x-\varOmega _{1}t) ), \end{aligned}$$

20 $$\begin{aligned} m_{2}(x,t)&=M_{2}\mu _{2}(x,t)\nonumber \\&=M_{2}~(1+A_{2}~\mathrm {sin}(\gamma _{2}x-\varOmega _{2}t) ), \end{aligned}$$

where $M_{1}>0$ and $M_{2}>0$ are the constant mass of the upper and lower rivulets, respectively. $A_{1}>0$ and $A_{2}>0$ are small parameters, and $\gamma _{1}>0$, $\gamma _{2}>0$, $\varOmega _{1}>0$, $\varOmega _{2}>0$. If $M_{0}>0$ is the constant mass of the beam cable, the total mass can be given by:

21 $$\begin{aligned} M(x,t)= & {} M\mu (x,t)=M(1+\bar{A}_{1}~\mathrm {sin}(\gamma _{1}x-\varOmega _{1}t)\nonumber \\&\quad +\bar{A}_{2}~\mathrm {sin}(\gamma _{2}x-\varOmega _{2}t) ), \end{aligned}$$

where $$M=M_{0}+M_{1}+M_{2}>0$$, $$\bar{A}_{1}=M_{1}A_{1}/M$$, and $$\bar{A}_{2}=M_{2}A_{2}/M$$.

The quasi-steady drag $c_{\mathcal {D}}(x,t;\phi _{i}^{*})$ and lift $c_{\mathcal {L}}(x,t;\phi _{i}^{*})$ coefficients may be obtained from wind-tunnel measurements. A quasi-steady assumption can be used due to the low-frequency oscillations of the cable. Yamaguchi shows the experimental results for the drag, lift and moment coefficients at various angles of wind attack with the ratio of diameters of rivulet and cable in [27]. These results show that the drag, lift and moment coefficients for different ratios of diameters of rivulet and cable are similar to each other. Due to these results, we define that the drag and lift coefficients may be written in periodic form in a small $\alpha _{0}, \beta _{0}$ and $\gamma _{0}$ neighbourhood of fixed $``\text {rivulet}''$ profiles as follows:

22 $$\begin{aligned} c_{\mathcal {D}}(x,t;\phi _{i}^{*})&= c_{\mathcal {D}} \Big [ \kappa _{1} \mu _{1}(x,t)+\kappa _{2} \mu _{2}(x,t)\Big ]\, , \end{aligned}$$

23 $$\begin{aligned} c_{\mathcal {L}}(x,t;\phi _{i}^{*})&= {c_{\mathcal {L}_{1}}} \Big [ (\phi _{1}^{*}(t)-\alpha _{1})\bar{\kappa _{1}}\mu _{1}(x,t)\nonumber \\&\quad +(\phi _{2}^{*}(t)-\alpha _{2})\bar{\kappa _{2}}\mu _{2}(x,t) \Big ]\nonumber \\&\quad +{c_{\mathcal {L}_{3}}} \Big [ (\phi _{1}^{*}(t)-\alpha _{1})^{3}{\bar{\bar{\kappa _{1}}}}\mu _{1}(x,t)\nonumber \\&\quad +(\phi _{2}^{*}(t)-\alpha _{2})^{3}{\bar{\bar{\kappa _{2}}}}\mu _{2}(x,t)\Big ] \, . \end{aligned}$$

Here $\kappa _{i}$, $\bar{\kappa _{i}}$ and $\bar{\bar{\kappa _{i}}}$ are constants for $i=1,2$ indicating the effect of the mass change of the rivulets on the drag and lift coefficients [17], and

24 $$\begin{aligned} c_{\mathcal {D}}= & {} \hat{c_{\mathcal {D}}}(1+\alpha _{0} ~\mathrm {sin}(\gamma _{0}x-\varOmega _{0}t)), \end{aligned}$$

25 $$\begin{aligned} c_{\mathcal {L}_{1}}= & {} \hat{c_{\mathcal {L}_{1}}}(1+\beta _{0} ~\mathrm {sin}(\gamma _{0}x-\varOmega _{0}t)), \end{aligned}$$

26 $$\begin{aligned} c_{\mathcal {L}_{3}}= & {} \hat{c_{\mathcal {L}_{3}}}(1+\sigma _{0} ~\mathrm {sin}(\gamma _{0}x-\varOmega _{0}t)), \end{aligned}$$

where $\alpha _{0}$, $\beta _{0}$ and $\sigma _{0}$ are small parameters, and $\gamma _{0}>0$, $\varOmega _{0}>0$. By neglecting terms of degree four and higher terms, we obtain the in-plane wind force as follows:

27 $$\begin{aligned} F_{y}&\approx -{\frac{\rho _{a}}{2}} d L v_{\infty }^2 \Big \{ A_{00}+ \frac{v_{t}}{v_{\infty }} A_{10}+ \frac{w_{t}}{v_{\infty }} A_{01}\nonumber \\&\quad +\frac{v_{t}^2}{v_{\infty }^2} A_{20}+ \frac{v_{t}w_{t}}{v_{\infty }^2} A_{11} \nonumber \\&\quad + \frac{w_{t}^2}{v_{\infty }^2} A_{02}+ \frac{v_{t}^3}{v_{\infty }^3} A_{30}+ \frac{v_{t}^2 w_{t}}{v_{\infty }^3} A_{21}\nonumber \\&\quad + \frac{v_{t} w_{t}^2}{v_{\infty }^3} A_{12}+ \frac{w_{t}^3}{v_{\infty }^3} A_{03} \Big \}, \end{aligned}$$

and similarly, we compute the out-of-plane wind force as follows:

28 $$\begin{aligned} F_{z}&\approx {\frac{\rho _{a}}{2}} d L v_{\infty }^2 \Big \{ B_{00}+ \frac{v_{t}}{v_{\infty }} B_{10}+ \frac{w_{t}}{v_{\infty }} B_{01}\nonumber \\&\quad +\frac{v_{t}^2}{v_{\infty }^2} B_{20}+ \frac{v_{t}w_{t}}{v_{\infty }^2} B_{11} \nonumber \\&\quad + \frac{w_{t}^2}{v_{\infty }^2} B_{02}+ \frac{v_{t}^3}{v_{\infty }^3} B_{30}+ \frac{v_{t}^2 w_{t}}{v_{\infty }^3} B_{21}\nonumber \\&\quad + \frac{v_{t} w_{t}^2}{v_{\infty }^3} B_{12}+ \frac{w_{t}^3}{v_{\infty }^3} B_{03} \Big \}, \end{aligned}$$

where $A_{ij}$ and $B_{ij}$ for $i,j=0,1,2,3$ as follows:

29 $$\begin{aligned} A_{ij}&= c_{\mathcal {D}} \Big [ \kappa _{1} \mu _{1}(x,t)+\kappa _{2} \mu _{2}(x,t)\Big ] a_{ij0} \nonumber \\&\quad +c_{\mathcal {L}_{1}} \Big [\bar{\kappa _{1}}\mu _{1}(x,t) a_{ij1}+ \bar{\kappa _{2}}\mu _{2}(x,t)a_{ij2} \Big ] \nonumber \\&\quad +c_{\mathcal {L}_{3}} \Big [{\bar{\bar{\kappa _{1}}}}\mu _{1}(x,t)a_{ij3}+{\bar{\bar{\kappa _{2}}}}\mu _{2}(x,t)a_{ij4} \Big ], \end{aligned}$$

and

30 $$\begin{aligned} B_{ij}&= c_{\mathcal {D}} \Big [ \kappa _{1} \mu _{1}(x,t)+\kappa _{2} \mu _{2}(x,t)\Big ] b_{ij0} \nonumber \\&\quad +c_{\mathcal {L}_{1}} \Big [\bar{\kappa _{1}}\mu _{1}(x,t) b_{ij1}+ \bar{\kappa _{2}}\mu _{2}(x,t)b_{ij2} \Big ] \nonumber \\&\quad +c_{\mathcal {L}_{3}} \Big [{\bar{\bar{\kappa _{1}}}}\mu _{1}(x,t)b_{ij3}+{\bar{\bar{\kappa _{2}}}}\mu _{2}(x,t)b_{ij4} \Big ]. \end{aligned}$$

The detailed expressions of the $a_{ijk}$- and $b_{ijk}$-coefficients for $k=1,2,3,4$ can be found in “Appendix A”. When we assume only in-plane horizontal cable motion with only the upper rivulet present, that is, $\gamma (\alpha ,\beta )=0$, $\mu _{2}(x,t)=0$, and $\kappa _{1}=\bar{\kappa _{1}}=\bar{\bar{\kappa _{1}}}=\mu _{1}(x,t)=1$, the same coefficients of $c_{\mathcal {D}}$, $c_{\mathcal {L}_{1}}$ and $c_{\mathcal {L}_{3}}$ are obtained as in [26].

## Further simplifications

For further calculations, we only consider in-plane motion ($w=0$) of the inclined beam, and we assume that there is only one rivulet on the cable, that is, $\gamma _{1}\approx \gamma _{2}$, $\omega _{1}\approx \omega _{2}$. Thus, we define $M(x,t)=M(1+\tilde{A}\mathrm {sin}(\gamma _{1}x-\varOmega _{1}t) )$, where $\tilde{A}$ is a small parameter. We substitute Eqs. (27) and (28), into Eqs. (10)–(11) and rewrite in order to obtain the equations of motion for the beam with a time-varying mass, yielding

31 $$\begin{aligned}&u_{tt} -\frac{E}{M}~ \frac{\partial }{\partial x} \Big (u_{x}+\frac{v_{x}^2}{2} \Big )= \Big [ \tilde{A}~\varOmega _{1}~\mathrm {cos}(\gamma _{1}x-\varOmega _{1}t)\Big ]u_{t}\nonumber \\&\qquad \qquad \qquad \qquad \qquad \qquad \qquad -\Big [ \tilde{A}\mathrm {sin}(\gamma _{1}x-\varOmega _{1}t)\Big ]u_{tt} \end{aligned}$$

32 $$\begin{aligned}&{ E I_{y} \over A M } v_{xxxx} -\frac{T_{0}}{AM}v_{xx}+v_{tt} -\frac{E}{M} \frac{\partial }{\partial x} \Big [ v_{x} \Big ( u_{x}+\frac{v_{x}^{2}}{2}\Big ) \Big ]\nonumber \\&\quad = \Big [ \tilde{A}~\varOmega _{1}~\mathrm {cos}(\gamma _{1}x-\varOmega _{1}t) \Big ]v_{t} \nonumber \\&\qquad -\Big [ \tilde{A}~\mathrm {sin}(\gamma _{1}x-\varOmega _{1}t) \Big ]v_{tt}\nonumber \\&\qquad -\Big [ 1+\tilde{A}~\mathrm {sin}(\gamma _{1}x-\varOmega _{1}t) \Big ] g ~\mathrm {sin}(\alpha ) v_{x} \nonumber \\&\qquad + \Big [(L-x)+\frac{ \tilde{A}}{\gamma _{1}}~\mathrm {cos}(\gamma _{1}x-\varOmega _{1}t)\nonumber \\&\qquad -\frac{ \tilde{A}}{\gamma _{1}}~\mathrm {cos}(\gamma _{1}L-\varOmega _{1}t)\Big ] g ~\mathrm {sin}(\alpha ) v_{xx} \nonumber \\&\qquad + \Big [1+ \tilde{A}~\mathrm {sin}(\gamma _{1}x-\varOmega _{1}t) \Big ] g ~\mathrm {cos}(\alpha ) \nonumber \\&\qquad -{\frac{\rho _{a}}{2AM}} d L v_{\infty }^2 \Big \{ A_{00}{+} \frac{v_{t}}{v_{\infty }} A_{10}{+} \frac{v_{t}^2}{v_{\infty }^2} A_{20}{+} \frac{v_{t}^3}{v_{\infty }^3} A_{30} \Big \}, \end{aligned}$$

where $A_{ij}$ for $i,j=0,1,2,3$ are defined in Eq. (29). As can be seen in Fig. 1, the inclined cable is attached to a sliding damper at $x=L$ and to a pylon at $x=0$. The boundary conditions are given by:

33 $$\begin{aligned} {u}(0,t)= & {} E I_{y}{v}_{xx}(0,t)={v}(0,t)={u}(L,t)\nonumber \\= & {} E I_{y} {v}_{x}(L,t)=0, \end{aligned}$$

and

34 $$\begin{aligned} E I_{y} {v}_{xxx}(L,t)= T_{0} {v}_{x}(L,t)+\lambda {v}_{t}(L,t), \end{aligned}$$

and the initial conditions are

35 $$\begin{aligned} {u}(x,0)&=u_{0}(x),~ {u}_{t}(x,0)=u_{1}(x), \end{aligned}$$

36 $$\begin{aligned} {v}(x,0)&=v_{0}(x),~ {v}_{t}(x,0)=v_{1}(x). \end{aligned}$$

Equations (31)–(32) represent the in-plane motion of the inclined beam cable system in the longitudinal and transversal direction, that is, in *x*-, and *y*-direction. We introduce the new variables $\bar{u}(x,t)$ and $\bar{v}(x,t)$ as defined by:

37 $$\begin{aligned} u(x,t)&=\bar{u}(x,t)+\hat{u}(x), \end{aligned}$$

38 $$\begin{aligned} v(x,t)&=\bar{v}(x,t)+\hat{v}(x), \end{aligned}$$

where $\hat{u}(x)$ and $\hat{v}(x)$ are the $``\text {stationary}''$ solutions by neglecting small-time perturbation (see “Appendix B”). When we substitute the new variables Eqs. (37)–(38) into the Eqs. (31)–(32), we obtain

39 $$\begin{aligned}&\bar{u}_{tt} -\frac{E}{M}~ \frac{\partial }{\partial x} \Big ( ~\bar{u}_{x}+\bar{v}_{x}\hat{v}_{x}+\frac{\bar{v}_{x}^2}{2} \Big )\nonumber \\&\quad =\Big [ \tilde{A}\varOmega _{1}\mathrm {cos}(\gamma _{1}x-\varOmega _{1}t)\Big ]\bar{u}_{t} \nonumber \\&\qquad -\Big [\tilde{A}\mathrm {sin}(\gamma _{1}x-\varOmega _{1}t)\Big ]\bar{u}_{tt} \end{aligned}$$

40 $$\begin{aligned}&{ E I_{y} \over A M } \bar{v}_{xxxx} -\frac{T_{0}}{AM}\bar{v}_{xx}+\bar{v}_{tt} \nonumber \\&\qquad -\frac{E}{M} \frac{\partial }{\partial x} \Big [ (\bar{v}_{x}+\hat{v}_{x})\Big ( \bar{u}_{x}+ \bar{v}_{x}\hat{v}_{x} +\frac{\bar{v}_{x}^{2}}{2}\Big )\nonumber \\&\qquad +\bar{v}_{x} \Big ( \hat{u}_{x}+\frac{\hat{v}_{x}^{2}}{2}\Big ) \Big ] \nonumber \\&\quad = \Big [ \tilde{A}~\varOmega _{1}\mathrm {cos}(\gamma _{1}x-\varOmega _{1}t) \Big ]\bar{v}_{t} \nonumber \\&\qquad -\Big [ \tilde{A}~\mathrm {sin}(\gamma _{1}x-\varOmega _{1}t) \Big ]\bar{v}_{tt} \nonumber \\&\qquad -\Big [ \tilde{A}~\mathrm {sin}(\gamma _{1}x\!-\!\varOmega _{1}t) \Big ] g ~\mathrm {sin}(\alpha ) (\bar{v}_{x}\!+\!\hat{v}_{x})\!-\!g ~\mathrm {sin}(\alpha ) \bar{v}_{x} \nonumber \\&\qquad + \Big [\frac{ \tilde{A}}{\gamma _{1}}~\mathrm {cos}(\gamma _{1}x-\varOmega _{1}t)\nonumber \\&\qquad -\frac{ \tilde{A}}{\gamma _{1}}~\mathrm {cos}(\gamma _{1}L-\varOmega _{1}t)\Big ] g ~\mathrm {sin}(\alpha ) (\bar{v}_{xx}+\hat{v}_{xx}) \nonumber \\&\qquad +(L\!-\!x)g ~\mathrm {sin}(\alpha ) \bar{v}_{xx} \!+\! \Big [ \tilde{A}~\mathrm {sin}(\gamma _{1}x\!-\!\varOmega _{1}t) \Big ] g ~\mathrm {cos}(\alpha ) \nonumber \\&\qquad -{\frac{\rho _{a}}{2AM}} d L v_{\infty }^2 \Big \{ \frac{\bar{v}_{t}}{v_{\infty }} A_{10}+ \frac{\bar{v}_{t}^2}{v_{\infty }^2} A_{20}+ \frac{\bar{v}_{t}^3}{v_{\infty }^3} A_{30} \Big \}. \end{aligned}$$

These coupled partial differential equations can be reduced to a single partial differential equation by applying Kirchhoff’s approximation. It will be assumed that $\hat{u}$ and $\bar{u}$ are $\mathcal {O}(\epsilon ^2)$, $\hat{v}$ and $\bar{v}$ are $\mathcal {O}(\epsilon )$, $\tilde{A}$ is $\mathcal {O}(\epsilon )$, $g \mathrm {sin(\alpha )}=P_{0}^{*}$ is $\mathcal {O}(1)$, and $\frac{E}{M}=P_{1}^{*}$ is $\mathcal {O}({1}/{\epsilon })$, where $\epsilon$ is a small parameter with $0<\epsilon \ll 1$. Then, by using these assumptions, Eq. (39) up to order $\epsilon$ becomes

41 $$\begin{aligned} -P_{1}^{*} \frac{\partial }{\partial x} \Big ( ~\bar{u}_{x}+\bar{v}_{x}\hat{v}_{x}+\frac{\bar{v}_{x}^2}{2} \Big )=0 \end{aligned}$$

First, we integrate Eq. (41) with respect to *x* from 0 to *x*, yielding

42 $$\begin{aligned} -P_{1}^{*} \Big ( ~\bar{u}_{x}+\bar{v}_{x}\hat{v}_{x}+\frac{\bar{v}_{x}^2}{2} \Big )= -h(t) \end{aligned}$$

and then from 0 to *L*, obtaining

43 $$\begin{aligned}&P_{1}^{*}\Big [ \bar{u}(L,t)-\bar{u}(0,t)+ \int _{0}^{L} \Big (\bar{v}_{x} \hat{v}_{x}+\frac{\bar{v}_{x}^2}{2}\Big ) \mathrm {d}x \Big ]\nonumber \\&\quad = \int _{0}^{L}h(t)\mathrm {d}x \end{aligned}$$

Hence,

44 $$\begin{aligned} h(t)=&\frac{P_{1}^{*}}{L}\Big [ \bar{u}(L,t)-\bar{u}(0,t)+ \int _{0}^{L} \Big (\bar{v}_{x} \hat{v}_{x}+\frac{\bar{v}_{x}^2}{2}\Big ) \mathrm {d}x \Big ] \end{aligned}$$

When we substitute Eq. (44) into Eq. (42), we obtain

45 $$\begin{aligned} \Big ( ~\bar{u}_{x}+\bar{v}_{x}\hat{v}_{x}+\frac{\bar{v}_{x}^2}{2} \Big )&= \frac{1}{L}\Big [ \bar{u}(L,t)-\bar{u}(0,t)\nonumber \\&\quad + \int _{0}^{L} \Big (\bar{v}_{x} \hat{v}_{x}+\frac{\bar{v}_{x}^2}{2}\Big ) \mathrm {d}x \Big ] \end{aligned}$$

Similarly, the equation for *v* in Eq. (40) can be rewritten in

46 $$\begin{aligned}&P_{2}^{*} \bar{v}_{xxxx} -P_{3}^{*}\bar{v}_{xx}+\bar{v}_{tt}\nonumber \\&\quad -P_{1}^{*} \frac{\partial }{\partial x} \Big [ (\bar{v}_{x}+\hat{v}_{x})\Big ( \bar{u}_{x} +\bar{v}_{x}\hat{v}_{x}+\frac{\bar{v}_{x}^{2}}{2}\Big )\nonumber \\&\quad +\bar{v}_{x} \Big ( \hat{u}_{x}+\frac{\hat{v}_{x}^{2}}{2}\Big ) \Big ]\nonumber \\&\quad = \Big [ \tilde{A}~\varOmega _{1}\mathrm {cos}(\gamma _{1}x-\varOmega _{1}t) \Big ]\bar{v}_{t}\nonumber \\&\quad -\Big [ \tilde{A}~\mathrm {sin}(\gamma _{1}x-\varOmega _{1}t) \Big ]\bar{v}_{tt} \nonumber \\ {}&\quad -\Big [ \tilde{A}~\mathrm {sin}(\gamma _{1}x-\varOmega _{1}t) \Big ] P_{0}^{*} (\bar{v}_{x}+\hat{v}_{x})-P_{0}^{*} \bar{v}_{x} \nonumber \\&\quad + \Big [\frac{ \tilde{A}}{\gamma _{1}}~\mathrm {cos}(\gamma _{1}x-\varOmega _{1}t) \nonumber \\&\quad -\frac{ \tilde{A}}{\gamma _{1}}~\mathrm {cos}(\gamma _{1}L-\varOmega _{1}t)\Big ] P_{0}^{*} (\bar{v}_{xx}+\hat{v}_{xx}) \nonumber \\&\quad +(L-x)P_{0}^{*} \bar{v}_{xx} + \Big [ \tilde{A}~\mathrm {sin}(\gamma _{1}x-\varOmega _{1}t) \Big ] P_{4}^{*} \nonumber \\&\quad -{\frac{\rho _{a}}{2AM}} d L v_{\infty }^2 \Big \{ \frac{\bar{v}_{t}}{v_{\infty }} A_{10}+ \frac{\bar{v}_{t}^2}{v_{\infty }^2} A_{20}+ \frac{\bar{v}_{t}^3}{v_{\infty }^3} A_{30} \Big \}. \end{aligned}$$

where $P_{2}^{*}={ E I_{y} \over A M }$ is $\mathcal {O}({1}/{\epsilon })$, $P_{3}^{*}={ T_{0} \over A M }$ is $\mathcal {O}({1}/{\epsilon })$, and $P_{4}^{*}=g \mathrm {cos(\alpha )}$ is $\mathcal {O}({1})$ . Substituting $\Big ( \bar{u}_{x}+ \bar{v}_{x}\hat{v}_{x}+\frac{\bar{v}_{x}^{2}}{2}\Big )$ from Eq. (45) and $\Big ( \hat{u}_{x}+\frac{\hat{v}_{x}^{2}}{2}\Big )$ from Eq. (241) as given in “Appendix B” into Eq. (46) we obtain

47 $$\begin{aligned}&P_{2}^{*} \bar{v}_{xxxx} +\bar{v}_{tt}-P_{3}^{*}\bar{v}_{xx}\nonumber \\&\quad = \frac{P_{1}^{*}}{L} (\bar{v}_{xx} +\hat{v}_{xx})\Big [ \bar{u}(L,t)-\bar{u}(0,t)\nonumber \\&\qquad + \int _{0}^{L} \Big (\bar{v}_{x} \hat{v}_{x}+\frac{\bar{v}_{x}^2}{2} \mathrm {d}x \Big ) \Big ] \nonumber \\&\qquad \Big [\tilde{A}~\varOmega _{1}\mathrm {cos}(\gamma _{1}x-\varOmega _{1}t) \Big ]\bar{v}_{t} -\Big [ \tilde{A}~\mathrm {sin}(\gamma _{1}x-\varOmega _{1}t) \Big ]\bar{v}_{tt} \nonumber \\&\qquad -\Big [ \tilde{A}~\mathrm {sin}(\gamma _{1}x-\varOmega _{1}t) \Big ] P_{0}^{*} (\bar{v}_{x}+\hat{v}_{x})-P_{0}^{*} \bar{v}_{x} \nonumber \\&\qquad + \Big [\frac{ \tilde{A}}{\gamma _{1}}~\mathrm {cos}(\gamma _{1}x-\varOmega _{1}t) -\frac{ \tilde{A}}{\gamma _{1}}~\mathrm {cos}(\gamma _{1}L\nonumber \\&\qquad -\varOmega _{1}t)\Big ] P_{0}^{*} (\bar{v}_{xx}+\hat{v}_{xx}) \nonumber \\&\qquad + \Big [ P_{0}^{*} (L-x) +\frac{P_{1}^{*}}{2L} \int _{0}^{L} \hat{v}_{x}^{2} \mathrm {d}x \Big ]\bar{v}_{xx} \nonumber \\&\qquad + \Big [ \tilde{A}~\mathrm {sin}(\gamma _{1}x-\varOmega _{1}t) \Big ] P_{4}^{*} \nonumber \\&\qquad -{\frac{\rho _{a}}{2AM}} d L v_{\infty }^2 \Big \{ \frac{\bar{v}_{t}}{v_{\infty }} A_{10}+ \frac{\bar{v}_{t}^2}{v_{\infty }^2} A_{20}+ \frac{\bar{v}_{t}^3}{v_{\infty }^3} A_{30} \Big \}. \end{aligned}$$

In order to put the equations in a non-dimensional form, the following dimensionless quantities are used:

$$\begin{aligned}&x^{*}=\frac{x}{L},~ t^{*}=\frac{t}{L} \sqrt{\frac{T_{0}}{AM}},\\&\bar{v}^{*}(x^{*},t^{*})=\frac{\bar{v}(x,t)}{L},~\hat{v}^{*}(x^{*})=\frac{\hat{v}(x)}{L},\\&v_{0}^{*}(x^{*})=\frac{v_{0}(x)}{L}, \\&\gamma _{1}^{*}= \gamma _{1} L,~v_{1}^{*}(x^{*})=\sqrt{\frac{AM}{T_{0}}}v_{1}(x),\\&\varOmega _{1}^{*}=\varOmega _{1}L\sqrt{\frac{AM}{T_{0}}}. \end{aligned}$$

Then, Eq. (47) in a non-dimensional form becomes

48 $$\begin{aligned}&\mu \bar{v}_{xxxx} +\bar{v}_{tt}-\bar{v}_{xx} \nonumber \\&\quad =\bar{\eta }_{1} (\bar{v}_{xx} +\hat{v}_{xx})\Big [ \int _{0}^{1} \Big (\bar{v}_{x} \hat{v}_{x}+\frac{\bar{v}_{x}^2}{2} \mathrm {d}x \Big ) \Big ] \nonumber \\&\quad + \Big [ \tilde{A}~\varOmega _{1}~\mathrm {cos}(\gamma _{1}x-\varOmega _{1}t) \Big ]\bar{v}_{t}+\bar{\eta }_{10}\bar{v}_{t} \nonumber \\&\quad -\Big [ \tilde{A}~\mathrm {sin}(\gamma _{1}x-\varOmega _{1}t) \Big ]\bar{v}_{tt} +\bar{\eta }_{20}\bar{v}_{t}^2+\bar{\eta }_{30} \bar{v}_{t}^3 \nonumber \\&\quad -\Big [ \tilde{A}~\mathrm {sin}(\gamma _{1}x-\varOmega _{1}t) \Big ] \eta _{2}(\bar{v}_{x}+\hat{v}_{x})-\bar{\eta }_{2}\bar{v}_{x} \nonumber \\&\quad + \Big [ \tilde{A}~\mathrm {cos}(\gamma _{1}x-\varOmega _{1}t) -\tilde{A}~\mathrm {cos}(\gamma _{1}\nonumber \\&\quad -\varOmega _{1}t)\Big ] \frac{ \bar{\eta }_{2}}{\gamma _{1}}(\bar{v}_{xx}+\hat{v}_{xx}) \nonumber \\&\quad + \Big [\bar{\eta }_{2} (1-x) +\frac{\bar{\eta }_{1}}{2}\int _{0}^{1} \hat{v}_{x}^{2} \mathrm {d}x \Big ]\bar{v}_{xx} \nonumber \\&\quad +\Big [\tilde{A}~\mathrm {sin}(\gamma _{1}x-\varOmega _{1}t) \Big ] \bar{\eta }_{3}, \end{aligned}$$

with the boundary conditions

49 $$\begin{aligned}&\bar{v}(0,t)=\bar{v}_{xx}(0,t)=\bar{v}_{x}(1,t)=0, \end{aligned}$$

50 $$\begin{aligned}&\mu \bar{v}_{xxx}(1,t)= \bar{v}_{x}(1,t)+ \epsilon \tilde{\lambda } \bar{v}_{t}(1,t), \end{aligned}$$

and the initial conditions

51 $$\begin{aligned} \bar{v}(x,0)=v_{0}(x),~ \bar{v}_{t}(x,0)=v_{1}(x), \end{aligned}$$

where $\mu = \frac{E I_{y}}{T_{0}L^{2}}, ~ \lambda ^{*}=\frac{\lambda }{\sqrt{AMT_{0}}}$, $\bar{\eta }_{1}={\frac{EA}{T_{0}} }$ is $\mathcal {O}({1})$, $\bar{\eta }_{10}=-\frac{1}{2\sqrt{AMT_{0}}} {\rho _{a}dL^2v_{\infty } A_{10}}>0$ is $\mathcal {O}(\epsilon )$, $\bar{\eta }_{20}=-\frac{1}{2AM}{\rho _{a}d L^2 A_{20}}$ is $\mathcal {O}(\epsilon )$, $\bar{\eta }_{30}=-{\frac{1}{2AMv_{\infty }}}\sqrt{\frac{T_{0}}{AM}}\rho _{a}d$ $L^2 A_{30}$ is $\mathcal {O}(\epsilon )$, $\bar{\eta }_{2}={\frac{AML}{T_{0}}g \mathrm {sin}(\alpha )}$ is $\mathcal {O}(\epsilon )$, and $\bar{\eta }_{3}={\frac{AML}{T_{0}}g \mathrm {cos}(\alpha )}$ is $\mathcal {O}(\epsilon )$. The damping coefficient $\lambda$ is assumed to be of $\mathcal {O}({\epsilon })$, that is, $\lambda =\epsilon \tilde{\lambda }$. We also assume that $\bar{v}(x,t)=\epsilon v(x,t)$, $\bar{\eta }_{10}=\epsilon \eta _{10}$, $\bar{\eta }_{2}=\epsilon \eta _{2}$, $\bar{\eta }_{3}=\epsilon \eta _{3}$, and $\tilde{A}=\epsilon \sigma$. The asterisks indicating the dimensional quantities are omitted in Eqs. (48) through (51), and henceforth for convenience. Then, by using these assumptions, Eq. (48) up to order $\epsilon ^2$ becomes

52 $$\begin{aligned}&\mu {v}_{xxxx} +{v}_{tt}-{v}_{xx} \nonumber \\&\quad =\epsilon \Big \{ \Big [ \eta _{10}+\sigma ~\varOmega _{1}~\mathrm {cos}(\gamma _{1}x-\varOmega _{1}t) \Big ]{v}_{t} \nonumber \\&\qquad - \sigma ~\mathrm {sin}(\gamma _{1}x-\varOmega _{1}t) {v}_{tt} \nonumber \\&\qquad + \eta _{2} (1-x) {v}_{xx}-\eta _{2}{v}_{x} \nonumber \\&\qquad +\sigma ~\mathrm {sin}(\gamma _{1}x-\varOmega _{1}t) \eta _{3} \Big \},~~t>0,~0<x<1, \end{aligned}$$

with the boundary conditions

53 $$\begin{aligned} {v}(0,t;\epsilon )&={v}_{xx}(0,t;\epsilon )={v}_{x}(1,t;\epsilon )=0, \end{aligned}$$

54 $$\begin{aligned} \mu {v}_{xxx}(1,t;\epsilon )&= {v}_{x}(1,t;\epsilon )+ \epsilon \tilde{\lambda } {v}_{t}(1,t;\epsilon ), \end{aligned}$$

and the initial conditions

55 $$\begin{aligned} {v}(x,0;\epsilon )=v_{0}(x),~ {v}_{t}(x,0;\epsilon )=v_{1}(x). \end{aligned}$$

In the following section, the initial-boundary value problem Eqs. (52)–(55) will be studied further.

## Application of the two-timescales perturbation method

In this section, the initial-boundary value problem Eqs. (52)–(55) will be studied and an approximation of the solution of the initial-boundary value problem up to order $\epsilon$ will be constructed by using a two-timescales perturbation method. We assume that *v*(*x*, *t*) can be expanded in a formal power series in $\epsilon$, that is,

56 $$\begin{aligned} {v}(x,t;\epsilon )={v}_{0}(x,t)+\epsilon {v}_{1}(x,t)+\epsilon ^{2} {v}_{2}(x,t)+\cdots , \end{aligned}$$

where all ${v}_{i}(x,t)$ and their derivatives for $i=0,1,2,\cdots$ are $\mathcal {O}(1)$ on a time scale of order $\epsilon ^{-1}$. The approximation of the solution may have secular terms which are unbounded terms in time. In order to avoid these secular terms, we will apply the two-timescales perturbation method by introducing a slow time scale $\tau =\epsilon t$, and

57 $$\begin{aligned} {v}(x,t;\epsilon )={y}(x,t,\tau ;\epsilon ). \end{aligned}$$

The following transformations are needed for the time derivatives

58 $$\begin{aligned} {v}_{t}= & {} {y}_{t}+\epsilon {y}_{\tau }, \end{aligned}$$

59 $$\begin{aligned} {v}_{tt}= & {} {y}_{tt}+2 \epsilon {y}_{t \tau }+ \epsilon ^{2}{y}_{\tau \tau }. \end{aligned}$$

Substitution of Eqs. (57)–(59) into Eq. (52) yields:

60 $$\begin{aligned}&\mu {y}_{xxxx} +{y}_{tt}-{y}_{xx} =\epsilon \Big \{ -2{y}_{t \tau }\nonumber \\&\quad +\Big [ \eta _{10}+\sigma ~\varOmega _{1}~\mathrm {cos}(\gamma _{1}x-\varOmega _{1}t) \Big ]{y}_{t} \nonumber \\&\quad - \sigma ~\mathrm {sin}(\gamma _{1}x-\varOmega _{1}t) {y}_{tt} \nonumber \\&\quad + \eta _{2} (1-x) {y}_{xx}-\eta _{2}{y}_{x} \nonumber \\&\quad +\sigma ~\mathrm {sin}(\gamma _{1}x-\varOmega _{1}t) \eta _{3} \Big \}+\mathcal {O}(\epsilon ^2), \end{aligned}$$

with the boundary conditions

61 $$\begin{aligned} {y}(0,t,\tau ;\epsilon )&={y}_{xx}(0,t,\tau ;\epsilon )={y}_{x}(1,t,\tau ;\epsilon )=0, \end{aligned}$$

62 $$\begin{aligned} \mu {y}_{xxx}(1,t,\tau ;\epsilon )&= {y}_{x}(1,t,\tau ;\epsilon ) + \epsilon [\tilde{\lambda }( {y}_{t}(1,t,\tau ;\epsilon )\nonumber \\&\quad +\epsilon {y}_{\tau }(1,t,\tau ;\epsilon ))], \end{aligned}$$

and the initial conditions

63 $$\begin{aligned} {y}(x,0,0;\epsilon )= & {} v_{0}(x),~ {y}_{t}(x,0,0;\epsilon )\nonumber \\&\quad +\,\epsilon {y}_{\tau }(x,0,0;\epsilon )=v_{1}(x). \end{aligned}$$

Assuming that

64 $$\begin{aligned} {y}(x,t,\tau ;\epsilon )= & {} {y}_{0}(x,t,\tau ;\epsilon )+\epsilon {y}_{1}(x,t,\tau ;\epsilon )\nonumber \\&\quad +\,\epsilon ^{2} {y}_{2}(x,t,\tau ;\epsilon )+\cdots , \end{aligned}$$

then by collecting terms of equal powers in $\epsilon$, it follows from the problem for ${y}(x,t,\tau ;\epsilon )$ that the $\mathcal {O}(1)$-problem is

65 $$\begin{aligned} \mu {y_0}_{xxxx} +{y_0}_{tt}-{y_0}_{xx}=&0, \end{aligned}$$

66 $$\begin{aligned} {y_0}(0,t,\tau )={y_0}_{xx}(0,t,\tau )=&0, \end{aligned}$$

67 $$\begin{aligned} {y_0}_{x}(1,t,\tau )=&0, \end{aligned}$$

68 $$\begin{aligned} \mu {y_0}_{xxx}(1,t,\tau )- {y_0}_{x}(1,t,\tau )=&0, \end{aligned}$$

69 $$\begin{aligned} {y_0}(x,0,0)=v_{0}(x),~\text {and}~ {y_0}_{t}(x,0,0)=&v_{1}(x), \end{aligned}$$

and that the $\mathcal {O}(\epsilon )$-problem is

70 $$\begin{aligned}&\mu {y_1}_{xxxx} +{y_1}_{tt}-{y_1}_{xx}=-2{y_0}_{t \tau }\nonumber \\&\quad +\Big [ \eta _{10} +\sigma ~\varOmega _{1}~\mathrm {cos}(\gamma _{1}x-\varOmega _{1}t) \Big ]{y_0}_{t} \nonumber \\&\quad -\sigma ~\mathrm {sin}(\gamma _{1}x-\varOmega _{1}t) {y_0}_{tt} + \eta _{2} (1-x) {y_0}_{xx}\nonumber \\&\quad -\eta _{2}{y_0}_{x}+ \sigma ~\mathrm {sin}(\gamma _{1}x-\varOmega _{1}t) \eta _{3}, \end{aligned}$$

71 $$\begin{aligned}&{y_1}(0,t,\tau )={y_1}_{xx}(0,t,\tau )=0, \end{aligned}$$

72 $$\begin{aligned}&{y_1}_{x}(1,t,\tau )=0, \end{aligned}$$

73 $$\begin{aligned}&\mu {y_1}_{xxx}(1,t,\tau )- {y_1}_{x}(1,t,\tau )= \tilde{\lambda } {y_0}_{t}(1,t,\tau ), \end{aligned}$$

74 $$\begin{aligned}&{y_1}(x,0,0)=0,~\text {and}~ {y_1}_{t}(x,0,0)+{y_0}_{\tau }(x,0,0)=0. \end{aligned}$$

The method of separation of variables will be applied to solve the problem Eqs. (65)–(69). The solution of the $\mathcal {O}(1)$-problem may be given in a special form

75 $$\begin{aligned} {y_0}(x,t,\tau )=T(t,\tau )\phi (x). \end{aligned}$$

By substitution of Eq. (75) into Eq. (65) and by dividing the so-obtained equation by $T(t,\tau )\phi (x)$ yields:

76 $$\begin{aligned} \frac{T_{tt}(t,\tau )}{T(t,\tau )}=\frac{\phi _{xx}(x)}{\phi (x)}-\mu \frac{\phi ^{(iv)}(x)}{\phi (x)}=-\omega . \end{aligned}$$

A separation constant is defined $-\omega$ so that the time-dependent part of the product solution oscillates for real and positive eigenvalues (for the proof, we refer the reader to [24]). We obtain a time-dependent part

77 $$\begin{aligned} {T_{tt}(t,\tau )}+\omega {T(t,\tau )}=0, \end{aligned}$$

and the general solution of the time-dependent part is a linear combination of sines and cosines in *t*,

78 $$\begin{aligned} {T(t,\tau )}=\sigma _{1}(\tau )\mathrm {cos}(\sqrt{\omega }t)+\sigma _{2}(\tau )\mathrm {sin}(\sqrt{\omega }t), \end{aligned}$$

where $\sigma _{1}$ and $\sigma _{2}$ are arbitrary function in $\tau$. In addition, we obtain a space-dependent part

79 $$\begin{aligned} \phi _{xxxx}(x)-\frac{1}{\mu }\phi _{xx}(x)-\frac{\omega }{\mu }\phi (x)=0, \end{aligned}$$

and the boundary conditions Eqs. (66)–(68) yield

80 $$\begin{aligned} \phi (0)=\phi _{xx}(0)=\phi _{x}(1)=\mu \phi _{xxx}(1)-\phi _{x}(1)=0. \end{aligned}$$

The characteristic equation for Eq. (79) is given by

81 $$\begin{aligned} m^4-\frac{m^2}{\mu }-\frac{\omega }{\mu }=0, \end{aligned}$$

and the solutions of Eq. (79) are given by

82 $$\begin{aligned} \phi (x)= & {} c_1 \mathrm {sinh}(ax)+c_2 \mathrm {cosh}(ax)+c_3 \mathrm {sin}(bx)\nonumber \\&\quad +\, c_4 \mathrm {cos}(bx), \end{aligned}$$

where $c_{i}$ for $i=1,2,3,4$ are constants, and $a=\sqrt{\frac{1+\sqrt{1+4\mu \omega }}{2\mu }}$ and $b=\sqrt{\frac{-1+\sqrt{1+4\mu \omega }}{2\mu }}$. The non-trivial solutions are found by using the boundary conditions Eq. (80), leading to the characteristic equation

83 $$\begin{aligned} f_{\mu }(\omega )= & {} -\mu ab\mathrm {cos}(b) \mathrm {cosh}(a)(a^2+b^2) \nonumber \\&\quad -\, a \mathrm {cosh}(a)\mathrm {sin}(b)\nonumber \\&\quad +\, b\mathrm {cos}(b)\mathrm {sinh}(a)=0. \end{aligned}$$

It follows from Eq. (83) that the eigenvalues $\omega _{n}=\mu b_{n}^4+b_{n}^2$ can be numerically computed for given values of $\mu$. The first ten eigenvalues $\omega _{n}$ are listed in Table 1. The eigenfunctions belonging to different eigenvalues are orthogonal with respect to the inner product; for details, the reader is referred to [24],

84 $$\begin{aligned} <\phi _{m}(x),\phi _{n}(x)>=\int _{0}^{1}\phi _{m}(x) \phi _{n}(x)\mathrm {d}{x}, \end{aligned}$$

and the eigenfunctions of the problem Eqs. (79)–(80) can be determined and are given by

85 $$\begin{aligned} \phi _{n}(x)=\theta _{n} \mathrm {sinh}(a_{n}x)+ \mathrm {sin}(b_{n}x), \end{aligned}$$

where $$\theta _{n}=-\frac{b_{n}\mathrm {cos}(b_{n})}{a_{n}\mathrm {cosh}(a_{n})}$$, $$a_{n}=\sqrt{\frac{1+\sqrt{1+4\mu \omega _{n}}}{2\mu }}$$, and $$b_{n}=\sqrt{\frac{-1+\sqrt{1+4\mu \omega _{n}}}{2\mu }}$$.

Hence, infinitely many non-trivial solutions of the initial-boundary problem Eqs. (65)–(69) have been determined. By using the superposition principle, the solution is obtained

86 $$\begin{aligned} {y_0}(x,t,\tau )= & {} \sum _{n=1}^{\infty } \Big [ A_{n}(\tau )\mathrm {cos}(\sqrt{\omega _{n}}t)\nonumber \\&\quad +B_{n}(\tau )\mathrm {sin}(\sqrt{\omega _{n}}t)\Big ]\phi _{n}(x). \end{aligned}$$

where $\phi _{n}(x)$ is given by Eq. (85), and where $A_{n}$ and $B_{n}$ are arbitrary functions in $\tau$ which can be used to avoid secular terms in $y_{1}(x,t,\tau )$. By using the initial conditions Eq. (69), we can determine $A_{n}(0)$ and $B_{n}(0)$, which are given by:

87 $$\begin{aligned} A_{n}(0)=\frac{1}{\zeta _{n}}\int _{0}^{1}v_{0}(x)\phi _{n}(x) \mathrm {d}{x}, \end{aligned}$$

and

88 $$\begin{aligned} \sqrt{\omega _{n}}B_{n}(0)=\frac{1}{\zeta _{n}}{\int _{0}^{1}v_{1}(x)\phi _{n}(x) \mathrm {d}{x}}, \end{aligned}$$

where

89 $$\begin{aligned} \zeta _{n}={\int _{0}^{1}\phi _{n}^2(x) \mathrm {d}{x}}. \end{aligned}$$

**Table 1:** Some eigenvalues $\omega _{i}$ which are roots of Eq. (83) with $\omega _{i}$ the *i*-th root

$\omega$	$\mu =0.001$	$\mu =0.01$	$\mu =0.1$	$\mu =1$
$\omega _{1}$	4.16497	4.30777	5.00501	10.54607
$\omega _{2}$	24.68972	29.20039	73.56205	517.34633
$\omega _{3}$	67.51921	101.78930	444.20353	3868.72929
$\omega _{4}$	137.55593	269.11062	1584.65381	14,740.35501
$\omega _{5}$	241.83773	601.31819	4196.24404	40,145.67525
$\omega _{6}$	389.72154	1191.92269	9214.09788	89,435.96197
$\omega _{7}$	592.89840	2157.81266	17,807.12342	174,300.30645
$\omega _{8}$	865.39593	3639.25581	31,378.01114	308,765.61727
$\omega _{9}$	1223.57912	5799.89869	51,563.23375	509,196.62255
$\omega _{10}$	1686.15056	8826.76635	80,233.04556	794,295.86667

Now, we solve the $\mathcal {O}(\epsilon )$-problem Eqs. (70)–(74). Due to having an inhomogeneous boundary condition Eq. (73), we use the following transformation to convert the problem into a problem with homogeneous boundary conditions

90 $$\begin{aligned} y_{1}(x,t,\tau )=V(x,t,\tau )+\Big ( \frac{x^{4}-2x^{3}}{12\mu +2} \Big )h(t,\tau ), \end{aligned}$$

where

91 $$\begin{aligned} h(t,\tau )=\tilde{\lambda } {y_0}_{t}(1,t,\tau ). \end{aligned}$$

Substituting Eq. (90) into Eqs. (70)–(74), we obtain

92 $$\begin{aligned}&\mu V_{xxxx} +V_{tt}-V_{xx}=-2{y_0}_{t \tau }\nonumber \\&\quad +\Big [ \eta _{10} +\sigma ~\varOmega _{1}~\mathrm {cos}(\gamma _{1}x-\varOmega _{1}t) \Big ]{y_0}_{t} -\eta _{2}{y_0}_{x} \nonumber \\&\quad - \sigma ~\mathrm {sin}(\gamma _{1}x-\varOmega _{1}t) {y_0}_{tt} + \eta _{2} (1-x) {y_0}_{xx} \nonumber \\&\quad +\sigma ~\mathrm {sin}(\gamma _{1}x-\varOmega _{1}t) \eta _{3} \nonumber \\&\quad +\Big ( \frac{12x^{2}-12x-24\mu }{12\mu +2} \Big )h(t,\tau )\nonumber \\&\quad -\Big ( \frac{x^{4}-2x^{3}}{12\mu +2} \Big )h_{tt}(t,\tau ), \end{aligned}$$

93 $$\begin{aligned}&{V}(0,t,\tau )={V}_{xx}(0,t,\tau )={V}_{x}(1,t,\tau )\nonumber \\&\quad -\Big ( \frac{2}{12\mu +2} \Big )h(t,\tau )=0, \end{aligned}$$

94 $$\begin{aligned}&\mu V_{xxx}(1,t,\tau )- {V}_{x}(1,t,\tau )=0, \end{aligned}$$

95 $$\begin{aligned}&V(x,0,0)=-\Big ( \frac{x^{4}-2x^{3}}{12\mu +2} \Big )h(0,0), \end{aligned}$$

96 $$\begin{aligned}&V_{t}(x,0,0)=-{y_0}_{\tau }(x,0,0)-\Big ( \frac{x^{4}-2x^{3}}{12\mu +2} \Big )h_{t}(0,0), \end{aligned}$$

where $h(t,\tau )$ is given by Eq. (91), and where *h*(0, 0), and $h_t(0,0)$ are given by

97 $$\begin{aligned} h(0,0)= & {} \tilde{\lambda } {v_1}(1), \end{aligned}$$

98 $$\begin{aligned} h_t(0,0)= & {} \tilde{\lambda } [{v_0}_{xx}(1)-\mu {v_0}_{xxxx}(1)]. \end{aligned}$$

In order to solve Eqs. (92)–(96), $V(x,t,\tau )$ is written in the following eigenfunction expansion

99 $$\begin{aligned} V(x,t,\tau )= \sum _{m=1}^{\infty }V_{m}(t,\tau )\phi _{m}(x), \end{aligned}$$

and by substituting Eq. (99) into the partial differential equation Eq. (92), we obtain

100 $$\begin{aligned}&\sum _{m=1}^{\infty } [V_{m_{tt}}(t,\tau ) +\omega _{m} V_{m}(t,\tau )]\phi _{m}(x)=-2{y_0}_{t \tau }\nonumber \\&\quad +\Big [ \eta _{10} +\sigma ~\varOmega _{1}~\mathrm {cos}(\gamma _{1}x-\varOmega _{1}t) \Big ]{y_0}_{t} -\eta _{2}{y_0}_{x} \nonumber \\&\quad - \sigma ~\mathrm {sin}(\gamma _{1}x-\varOmega _{1}t) {y_0}_{tt} + \eta _{2} (1-x) {y_0}_{xx} \nonumber \\&\quad +\sigma ~\mathrm {sin}(\gamma _{1}x-\varOmega _{1}t) \eta _{3}\nonumber \\&\quad +\Big ( \frac{12x^{2}-12x-24\mu }{12\mu +2} \Big )h(t,\tau ) \nonumber \\&\quad -\Big ( \frac{x^{4}-2x^{3}}{12\mu +2} \Big )h_{tt}(t,\tau ). \end{aligned}$$

We expand $\Big ( \frac{x^{4}-2x^{3}}{12\mu +2} \Big )$ and $\Big ( \frac{12x^{2}-12x-24\mu }{12\mu +2} \Big )$ into a series of eigenfunctions $\phi _{m}(x)$, and we obtain

101 $$\begin{aligned}&\Big ( \frac{x^{4}-2x^{3}}{12\mu +2} \Big )= \sum _{m=1}^{\infty }c_{m}\phi _{m}(x), \end{aligned}$$

102 $$\begin{aligned}&\Big ( \frac{12x^{2}-12x-24\mu }{12\mu +2} \Big )= \sum _{m=1}^{\infty }d_{m}\phi _{m}(x), \end{aligned}$$

where

103 $$\begin{aligned} c_{m}= & {} \frac{1}{\zeta _{m}}{\int _{0}^{1}\Big ( \frac{x^{4}-2x^{3}}{12\mu +2} \Big ) \phi _{m}(x) \mathrm {d}{x}}, \end{aligned}$$

104 $$\begin{aligned} d_{m}= & {} \frac{1}{\zeta _{m}}{\int _{0}^{1} \Big ( \frac{12x^{2}-12x-24\mu }{12\mu +2} \Big ) \phi _{m}(x) \mathrm {d}{x}}, \end{aligned}$$

and where $\zeta _{m}$ is given by Eq. (89). By multiplying both sides of Eq. (100) by $\phi _{n}(x)$, and then by integrating from $x=0$ to $x=1$, and by using the orthogonality properties of the eigenfunctions, we obtain

105 $$\begin{aligned}&\Big [V_{n_{tt}}(t,\tau ) +\omega _{n} V_{n}(t,\tau )\Big ]=-c_{n}h_{tt}(t,\tau )+d_{n}h(t,\tau )\nonumber \\&\quad -2{T_{n_{t\tau }}}(t,\tau ) \nonumber \\&\qquad + \frac{\eta _{2}}{\zeta _{n}}{T_{n}}(t,\tau )(\hat{\varPhi }_{nn}-{\varPhi _{nn}})\nonumber \\&\qquad +\frac{\eta _{2}}{\zeta _{n}} \sum _{\begin{array}{c} m=1 \\ m \ne n \end{array}}^{\infty } {T_{m}}(t,\tau )(\hat{\varPhi }_{mn}-{\varPhi _{mn}}) \nonumber \\&\qquad +\frac{\sigma ~\eta _{3}}{\zeta _{n}}\Big (\mathrm {cos}(\varOmega _{1}t){\hat{\varUpsilon }_{n}}-\mathrm {sin}(\varOmega _{1}t){\varUpsilon }_{n}\Big )+\eta _{10}{T_{n_{t}}}(t,\tau ) \nonumber \\&\qquad +\frac{\sigma ~\varOmega _{1}}{\zeta _{n}}{T_{n_{t}}}(t,\tau ) \Big (\mathrm {cos}(\varOmega _{1}t){\varPsi }_{nn}+\mathrm {sin}(\varOmega _{1}t){\hat{\varPsi }_{nn}} \Big ) \nonumber \\&\qquad +\frac{\sigma }{\zeta _{n}}{T_{n_{tt}}}(t,\tau ) \Big (\mathrm {sin}(\varOmega _{1}t){\varPsi }_{nn}-\mathrm {cos}(\varOmega _{1}t){\hat{\varPsi }_{nn}} \Big )\nonumber \\&\qquad +\frac{\sigma }{\zeta _{n}}\sum _{\begin{array}{c} m=1 \\ m \ne n \end{array}}^{\infty } \Big [ \varOmega _{1}{T_{m_{t}}}(t,\tau ) \Big (\mathrm {cos}(\varOmega _{1}t){\varPsi }_{mn}\nonumber \\&\qquad +\mathrm {sin}(\varOmega _{1}t){\hat{\varPsi }_{mn}} \Big ) \nonumber \\&\qquad +{T_{m_{tt}}}(t,\tau ) \Big (\mathrm {sin}(\varOmega _{1}t){\varPsi }_{mn}-\mathrm {cos}(\varOmega _{1}t){\hat{\varPsi }_{mn}} \Big ) \Big ], \end{aligned}$$

where

106 $$\begin{aligned} T_{m}(t,\tau )=A_{m}(\tau )\mathrm {cos}(\sqrt{\omega _{m}}t)+ B_{m}(\tau )\mathrm {sin}(\sqrt{\omega _{m}}t), \end{aligned}$$

and

107 $$\begin{aligned} \varPhi _{mn}&=\int _{0}^{1}\frac{\mathrm {d} \phi _{m}(x)}{\mathrm {d} x} \phi _{n}(x)\mathrm {d}{x}, \end{aligned}$$

108 $$\begin{aligned} \hat{\varPhi }_{mn}&=\int _{0}^{1}(1-x)\frac{\mathrm {d}^2 \phi _{m}(x)}{\mathrm {d} x^2}\phi _{n}(x)\mathrm {d}{x}, \end{aligned}$$

109 $$\begin{aligned} \varPsi _{mn}&=\int _{0}^{1}\mathrm {cos}(\gamma _{1}x)\phi _{m}(x)\phi _{n}(x)\mathrm {d}{x}, \end{aligned}$$

110 $$\begin{aligned} \hat{\varPsi }_{mn}&=\int _{0}^{1}\mathrm {sin}(\gamma _{1}x)\phi _{m}(x)\phi _{n}(x)\mathrm {d}{x}, \end{aligned}$$

111 $$\begin{aligned} \varUpsilon _{m}&=\int _{0}^{1}\mathrm {cos}(\gamma _{1}x)\phi _{m}(x)\mathrm {d}{x}, \end{aligned}$$

112 $$\begin{aligned} \hat{\varUpsilon }_{m}&=\int _{0}^{1}\mathrm {sin}(\gamma _{1}x)\phi _{m}(x)\mathrm {d}{x}. \end{aligned}$$

It follows from Eqs. (91) and (86) that $h(t,\tau )$ and $h_{tt}(t,\tau )$ can be written as

113 $$\begin{aligned} h(t,\tau )= & {} \tilde{\lambda } \sum _{m=1}^{\infty }T_{m_{t}}(t,\tau )\phi _{m}(1), \end{aligned}$$

114 $$\begin{aligned} h_{tt}(t,\tau )= & {} \tilde{\lambda } \sum _{m=1}^{\infty }T_{m_{ttt}}(t,\tau )\phi _{m}(1), \end{aligned}$$

Hence, by using Eqs. (113) and (114) it follows that Eq. (105) can be rewritten as

115 $$\begin{aligned}&\Big [V_{n_{tt}}(t,\tau ) +\omega _{n} V_{n}(t,\tau )\Big ]\nonumber \\&\quad =\sum _{\begin{array}{c} m=1 \\ m \ne n \end{array}}^{\infty } \Big \{(c_{n}\omega _{m}+d_{n})\tilde{\lambda }\phi _{m}(1)\sqrt{\omega _{m}}\times \nonumber \\&\quad \Big [-A_{m}(\tau ) \mathrm {sin}(\sqrt{\omega _{m}}t)\nonumber \\&\quad +B_{m}(\tau )\mathrm {cos}(\sqrt{\omega _{m}}t) \Big ] \Big \} \nonumber \\&\quad + \frac{\eta _{2}}{\zeta _{n}}\sum _{\begin{array}{c} m=1 \\ m \ne n \end{array}}^{\infty } (\hat{\varPhi }_{mn}-{\varPhi _{mn}}) \Big [ A_{m}(\tau )\mathrm {cos}(\sqrt{\omega _{m}}t)\nonumber \\ {}&\quad + B_{m}(\tau )\mathrm {sin}(\sqrt{\omega _{m}}t) \Big ] \nonumber \\&\quad +\mathrm {sin}(\sqrt{\omega _{n}}t) \Big \{ 2\sqrt{\omega _{n}}\frac{\mathrm {d} A_{n}(\tau )}{\mathrm {d} \tau }\nonumber \\&\quad -A_{n}(\tau ) \Big [\eta _{10}\sqrt{\omega _{n}}+\tilde{\lambda }\phi _{n}(1)\sqrt{\omega _{n}}(c_{n}\omega _{n}+d_{n}) \Big ] \nonumber \\&\quad +B_{n}(\tau ) \Big [\frac{\eta _{2}}{\zeta _{n}}(\hat{\varPhi }_{nn}-{\varPhi _{nn}}) \Big ] \Big \}\nonumber \\&\quad +\mathrm {cos}(\sqrt{\omega _{n}}t) \Big \{-2\sqrt{\omega _{n}}\frac{\mathrm {d} B_{n}(\tau )}{\mathrm {d} \tau }\nonumber \\&\quad +B_{n}(\tau ) \Big [\eta _{10}\sqrt{\omega _{n}}+\tilde{\lambda }\phi _{n}(1)\sqrt{\omega _{n}}(c_{n}\omega _{n}+d_{n}) \Big ] \nonumber \\&\quad +A_{n}(\tau ) \Big [\frac{\eta _{2}}{\zeta _{n}}(\hat{\varPhi }_{nn}-{\varPhi _{nn}}) \Big ] \Big \} \nonumber \\&\quad +\frac{\sigma ~\eta _{3}}{\zeta _{n}}\Big (\mathrm {cos}(\varOmega _{1}t){\hat{\varUpsilon }_{n}}-\mathrm {sin}(\varOmega _{1}t){\varUpsilon }_{n}\Big ) \nonumber \\&\quad +\mathrm {sin}(\varOmega _{1}t+\sqrt{\omega _{n}}t) \frac{\sigma }{2\zeta {n}} (\varOmega _{1}\sqrt{\omega _{n}}+\omega _{n})\times \nonumber \\&\quad \Big [-A_{n}(\tau ) {\varPsi }_{nn}+B_{n}(\tau ){\hat{\varPsi }_{nn}} \Big ] \nonumber \\&\quad +\mathrm {sin}(\varOmega _{1}t-\sqrt{\omega _{n}}t) \frac{\sigma }{2\zeta {n}} (\varOmega _{1}\sqrt{\omega _{n}}-\omega _{n})\times \nonumber \\&\quad \Big [A_{n}(\tau ) {\varPsi }_{nn}+B_{n}(\tau ){\hat{\varPsi }_{nn}} \Big ] \nonumber \\&\quad +\mathrm {cos}(\varOmega _{1}t+\sqrt{\omega _{n}}t) \frac{\sigma }{2\zeta {n}} (\varOmega _{1}\sqrt{\omega _{n}}+\omega _{n})\times \nonumber \\&\quad \Big [A_{n}(\tau ) \hat{\varPsi }_{nn}+B_{n}(\tau ){{\varPsi }_{nn}} \Big ] \nonumber \\&\quad +\mathrm {cos}(\varOmega _{1}t-\sqrt{\omega _{n}}t) \frac{\sigma }{2\zeta {n}} (\varOmega _{1}\sqrt{\omega _{n}}-\omega _{n})\times \nonumber \\&\quad \Big [-A_{n}(\tau ) \hat{\varPsi }_{nn}+B_{n}(\tau ){{\varPsi }_{nn}} \Big ] \nonumber \\&\quad +\frac{\sigma }{2\zeta _{n}}\sum _{\begin{array}{c} m=1 \\ m \ne n \end{array}}^{\infty } \Big \{ \mathrm {sin}(\varOmega _{1}t+\sqrt{\omega _{m}}t) (\varOmega _{1}\sqrt{\omega _{m}}+\omega _{m}) \times \nonumber \\&\quad \Big [-A_{m}(\tau ) {\varPsi }_{mn}+B_{m}(\tau ){\hat{\varPsi }_{mn}} \Big ] \nonumber \\&\quad +\mathrm {sin}(\varOmega _{1}t-\sqrt{\omega _{m}}t) (\varOmega _{1}\sqrt{\omega _{m}}-\omega _{m}) \times \nonumber \\&\quad \Big [A_{m}(\tau ) {\varPsi }_{mn}+B_{m}(\tau ){\hat{\varPsi }_{mn}} \Big ] \nonumber \\&\quad +\mathrm {cos}(\varOmega _{1}t+\sqrt{\omega _{m}}t) (\varOmega _{1}\sqrt{\omega _{m}}+\omega _{m}) \times \nonumber \\&\quad \Big [A_{m}(\tau ) \hat{\varPsi }_{mn}+B_{m}(\tau ){{\varPsi }_{mn}} \Big ] \nonumber \\&\quad +\mathrm {cos}(\varOmega _{1}t-\sqrt{\omega _{m}}t) (\varOmega _{1}\sqrt{\omega _{m}}-\omega _{m}) \times \nonumber \\&\quad \Big [-A_{m}(\tau ) \hat{\varPsi }_{mn}+B_{m}(\tau ){{\varPsi }_{mn}} \Big ] \Big \}. \end{aligned}$$

It can be easily seen from Eq. (115) that there are infinitely many values of $\varOmega _{1}$, which can cause internal resonances. The possible resonance cases are as follows:

(i) The non-resonant case: if $\varOmega _{1}$ is not within an order $\epsilon$-neighbourhood of the frequencies $\sqrt{\omega _{n}} \pm \sqrt{\omega _{m}}$ for all *m* and *n*.

(ii) The near resonance case (Resonance detuning): if $\varOmega _{1}$ is within an order $\epsilon$-neighbourhood of $\sqrt{\omega _{n}}\pm \sqrt{\omega _{m}}$ for certain fixed *m* and *n*.

(iii) The pure resonance case: if $\varOmega _{1} = \sqrt{\omega _{n}}+ \sqrt{\omega _{m}}$ for certain fixed *m* and *n*. This is the sum-type resonance case. If $\varOmega _{1} = \sqrt{\omega _{n}}- \sqrt{\omega _{m}}$ for certain fixed *m* and *n*, it is the difference-type resonance case.

In the following subsections, we will study the non-resonant case, the sum-type resonance case, the difference-type resonance case and by considering only some of the first few modes and omitting the higher-order modes.

For simplicity, we will now assume that $\varOmega _{1} = \sqrt{\omega _{1}}+ \sqrt{\omega _{2}}$ (or $\varOmega _{1} = \sqrt{\omega _{2}}- \sqrt{\omega _{1}}$). Resonance can occur when $\varOmega _{1} = \pm \sqrt{\omega _{n}}\pm \sqrt{\omega _{m}}$ for some *n* and *m*. We have to consider the following three cases:

(i) $\varOmega _{1} = - \sqrt{\omega _{n}}- \sqrt{\omega _{m}}$, which has no solution since the right-hand side is negative, while the left-hand side is positive.
(ii) $\varOmega _{1} = \sqrt{\omega _{n}}+ \sqrt{\omega _{m}}$, which has only the trivial solution $m=2$, $n=1$ or $m=1$, $n=2$.
(iii) $\varOmega _{1} = \sqrt{\omega _{n}}- \sqrt{\omega _{m}}$ (or equivalently $\varOmega _{1} = \sqrt{\omega _{m}}- \sqrt{\omega _{n}}$), which has only solutions by looking at Table 4.1 for certain values of $\mu$. There are possibilities that three modes are interacting. For example, $\sqrt{\omega _{1}}+ \sqrt{\omega _{2}} \cong \sqrt{\omega _{4}}- \sqrt{\omega _{2}}$ for $\mu =0.001$.

### The non-resonant case

When $\varOmega _{1} \ne \sqrt{\omega _{n}}\pm \sqrt{\omega _{m}}+\mathcal {O}(\epsilon )$ for all *m* and *n* only resonances occur due to the fourth and fifth term in the right-hand side of Eq. (115), that is, Eq. (115) can be rewritten as:

116 $$\begin{aligned}&\Big [V_{n_{tt}}(t,\tau ) +\omega _{n} V_{n}(t,\tau )\Big ]= \mathrm {sin}(\sqrt{\omega _{n}}t) \Big \{ 2\sqrt{\omega _{n}}\frac{\mathrm {d} A_{n}(\tau )}{\mathrm {d} \tau }\nonumber \\&\quad -A_{n}(\tau ) \Big [\eta _{10}\sqrt{\omega _{n}}+\tilde{\lambda }\phi _{n}(1)\sqrt{\omega _{n}}(c_{n}\omega _{n}+d_{n}) \Big ] \nonumber \\&\quad +B_{n}(\tau ) \Big [\frac{\eta _{2}}{\zeta _{n}}(\hat{\varPhi }_{nn}-{\varPhi _{nn}}) \Big ] \Big \}\nonumber \\&\quad +\mathrm {cos}(\sqrt{\omega _{n}}t) \Big \{-2\sqrt{\omega _{n}}\frac{\mathrm {d} B_{n}(\tau )}{\mathrm {d} \tau } \nonumber \\&\quad +B_{n}(\tau ) \Big [\eta _{10}\sqrt{\omega _{n}}+\tilde{\lambda }\phi _{n}(1)\sqrt{\omega _{n}}(c_{n}\omega _{n}+d_{n}) \Big ]\nonumber \\&\quad +A_{n}(\tau ) \Big [\frac{\eta _{2}}{\zeta _{n}}(\hat{\varPhi }_{nn}-{\varPhi _{nn}}) \Big ] \Big \} \nonumber \\&\quad +``\text {NST''}, \end{aligned}$$

where “NST” stands for terms that lead to nonsecular terms in $V_{n}$. In order to remove secular terms, it follows from Eq. (116) that $A_{n}(\tau )$ and $B_{n}(\tau )$ have to satisfy

117 $$\begin{aligned}&\frac{\mathrm {d} A_{n}(\tau )}{\mathrm {d} \tau }-A_{n}(\tau ) X_{n}+B_{n}(\tau ) Y_{n}=0, \end{aligned}$$

118 $$\begin{aligned}&\frac{\mathrm {d} B_{n}(\tau )}{\mathrm {d} \tau }-B_{n}(\tau ) X_{n}-A_{n}(\tau ) Y_{n}=0, \end{aligned}$$

where $X_n$ and $Y_n$ are defined by

119 $$\begin{aligned} X_{n}&=\Big [ \frac{\eta _{10}}{2}+\frac{\tilde{\lambda }}{2}\phi _{n}(1)(c_{n}\omega _{n}+d_{n}) \Big ], \end{aligned}$$

120 $$\begin{aligned} Y_{n}&= \frac{\eta _{2}}{2\zeta _{n}\sqrt{\omega _{n}}}(\hat{\varPhi }_{nn}-{\varPhi _{nn}}) . \end{aligned}$$

The solution of Eqs. (117) and (118) is given by

121 $$\begin{aligned} A_{n}(\tau )&=\Big [A_{n}(0) \mathrm {cos}(Y_{n}\tau ) -B_{n}(0) \mathrm {sin}(Y_{n}\tau ) \Big ]\mathrm {e}^{X_{n}\tau }, \end{aligned}$$

122 $$\begin{aligned} B_{n}(\tau )&=\Big [A_{n}(0) \mathrm {sin}(Y_{n}\tau ) +B_{n}(0) \mathrm {cos}(Y_{n}\tau ) \Big ]\mathrm {e}^{X_{n}\tau }, \end{aligned}$$

where $A_{n}(0)$ and $B_{n}(0)$ are given by Eqs. (87) and (88), respectively. By using Eqs. (103) and (104) with Eqs. (79) and (80) it can easily be shown that $\frac{1}{2}(c_{n}\omega _{n}+d_{n})=\frac{\mu }{\zeta _{n}(12\mu +2)}\frac{\mathrm {d}^2 \phi _{n}(1)}{\mathrm {d} x^2}-\frac{1}{2\zeta _{n}} \phi _{n}(1)$. Hence, Eq. (106) with Eq. (119) and Eq. (122) can be rewritten as:

123 $$\begin{aligned} T_{m}(t,\tau )= & {} \mathrm {e}^{X_{m}\tau } \Big [ A_{m}(0)\mathrm {cos}(\sqrt{\omega _{m}}t-Y_{m}\tau )\nonumber \\&\quad +B_{m}(0)\mathrm {sin}(\sqrt{\omega _{m}}t-Y_{m}\tau ) \Big ]. \end{aligned}$$

Now, from Eq. (116) with Eqs. (117)–(123), we can obtain $V_{n}(t,\tau )$ straightforwardly. Obviously, $V_{n}(t,\tau )$ will be bounded on a time scale of order ${1}/{\epsilon }$, and so will be $V_{n}(x,t,\tau )$.

**Table 2:** Numerical approximations of $\omega _{n}$ and $\tilde{p}_n=\frac{1}{2}\phi _{n}(1)(c_{n}\omega _{n}+d_{n})$ for different values of $\mu$

*n*	$\mu =0.001$		$\mu =0.01$		$\mu =0.1$		$\mu =1$	
	$\omega _n$	$\tilde{p}_n$	$\omega _n$	$\tilde{p}_n$	$\omega _n$	$\tilde{p}_n$	$\omega _n$	$\tilde{p}_n$
1	4.16497	$-$ 0.68935	4.30777	$-$ 0.74453	5.00501	$-$ 0.98509	10.54607	$-$ 1.31501
2	24.68972	$-$ 0.97241	29.20039	$-$ 1.20184	73.56205	$-$ 2.39752	517.34633	$-$ 4.17474
3	67.51921	$-$ 1.05857	101.78930	$-$ 1.59718	444.20353	$-$ 4.85921	3868.72929	$-$ 9.81249
4	137.55593	$-$ 1.12954	269.11062	$-$ 2.15236	1584.65381	$-$ 8.55772	14,740.35501	$-$ 18.27181
5	241.83773	$-$ 1.21100	601.31819	$-$ 2.89309	4196.24404	$-$ 13.49164	40,145.67525	$-$ 29.55129
6	389.72154	$-$ 1.30902	1191.92269	$-$ 3.82147	9214.09788	$-$ 19.65981	89,435.96197	$-$ 43.65071
7	592.89840	$-$ 1.42560	2157.81266	$-$ 4.93712	17,807.12342	$-$ 27.06189	174,300.30645	$-$ 60.57004
8	865.39593	$-$ 1.56152	3639.25581	$-$ 6.23961	31,378.01114	$-$ 35.69774	308,765.61727	$-$ 80.30926
9	1223.57912	$-$ 1.71708	5799.89869	$-$ 7.72870	51,563.23375	$-$ 45.56732	509,196.62255	$-$ 102.86836
10	1686.15056	$-$ 1.89241	8826.76635	$-$ 9.40424	80,233.04556	$-$ 56.67063	794,295.86667	$-$ 128.24735
25	41,022.73344	$-$ 6.88972	356,891.24613	$-$ 56.88899	3,515,576.38882	$-$ 371.26434	35,102,427.80053	$-$ 847.31857
50	609,002.39910	$-$ 25.03887	5,872,359.00977	$-$ 229.14149	58,505,925.11380	$-$ 1512.43738	584,841,586.15436	$-$ 3455.71402

In Table 2, numerical approximations of $\omega _n$ and the damping parameter $\tilde{p}_n$ are given for different values of $\mu$. For different bending stiffness, we can choose the damping parameter in such a way that all modes are damped by taking $\tilde{\lambda }$ appropriately compared to the $\eta _{10}$ coefficient. It is also clear to see from Table 2 that for smaller values of $\mu$, we should take larger $\tilde{\lambda }$ to have damping for all modes. In Table 3, it can be seen as expected that the bending stiffness $\mu$ and the damping parameter $\tilde{\lambda }$ influence the stability.

**Fig. 3:**
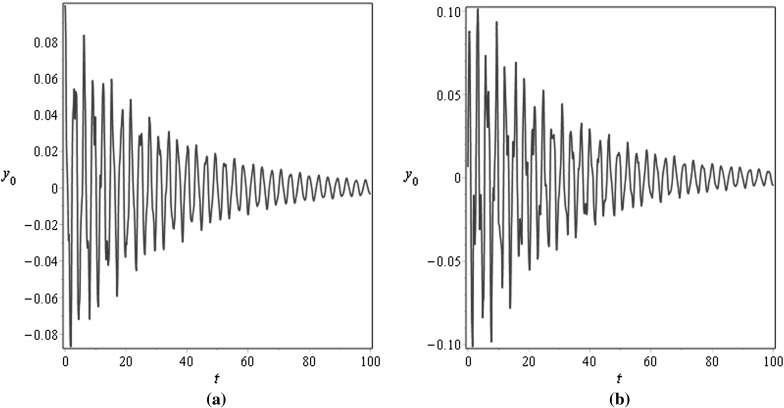
Transverse displacements $y_0$ at **a** $x=0.5$ and at **b** $x=1$ against time *t* with the initial displacement $v_0(x)=0.1\mathrm {sin}(\pi x)$ and the initial velocity $v_1(x)=0.05 \mathrm {sin}(\pi x)$ for $\mu =0.001$, $\eta _{10}=\eta _2=\epsilon =0.1$, $\lambda =0.5$, and $N=10$

**Fig. 4:**
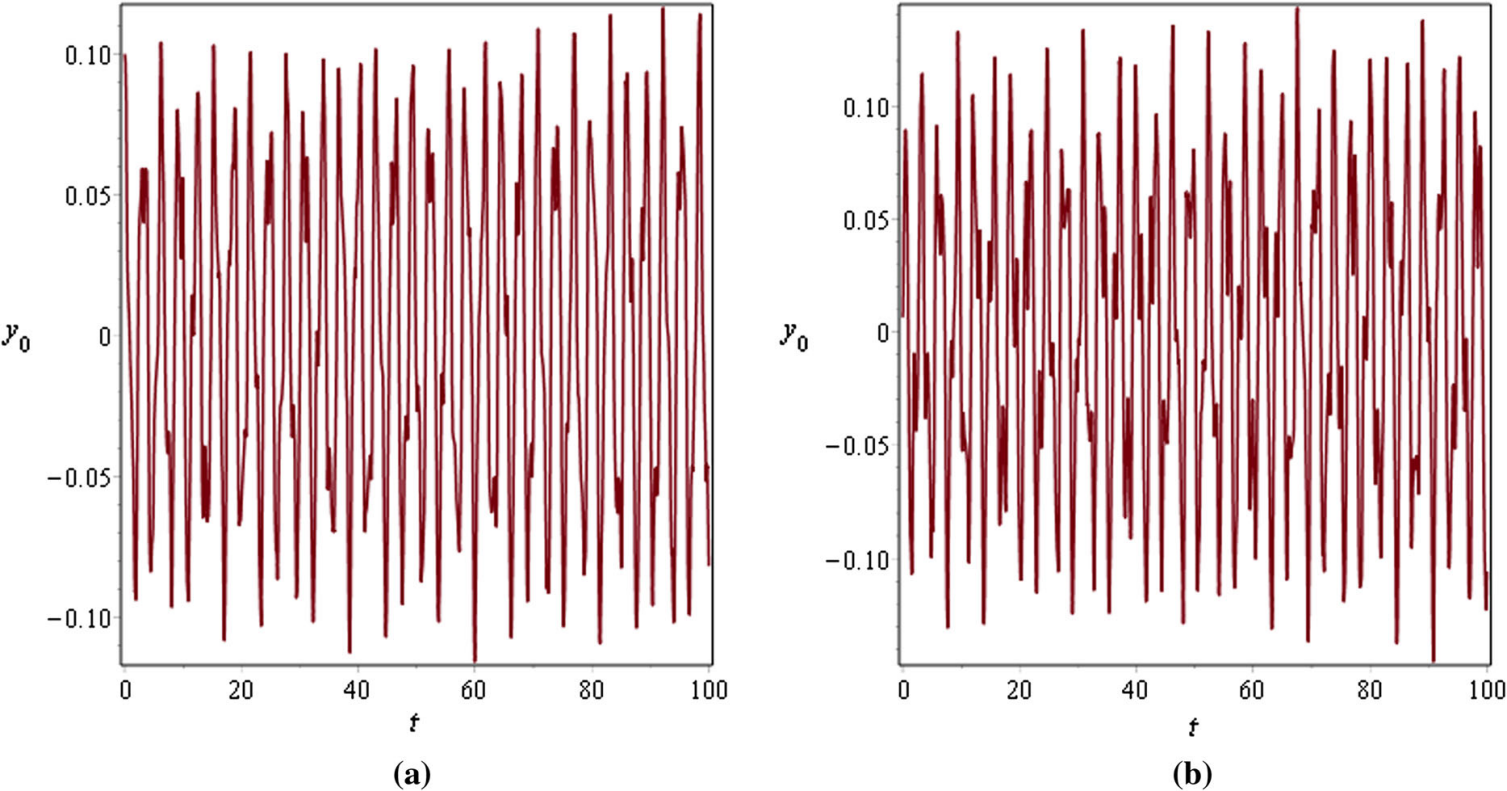
Transverse displacements $y_0$ at **a** $x=0.5$ and at **b** $x=1$ against time *t* with the initial displacement $v_0(x)=0.1\mathrm {sin}(\pi x)$ and the initial velocity $v_1(x)=0.05 \mathrm {sin}(\pi x)$ for $\mu =0.001$, $\eta _{10}=\eta _2=\epsilon =0.1$, $\lambda =0.05$, and $N=10$

**Fig. 5:**
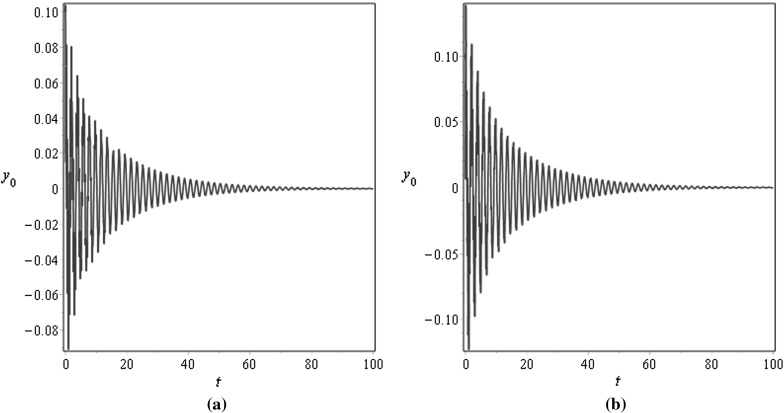
Transverse displacements $y_0$ at **a** $x=0.5$ and at **b** $x=1$ against time *t* with the initial displacement $v_0(x)=0.1\mathrm {sin}(\pi x)$ and the initial velocity $v_1(x)=0.05 \mathrm {sin}(\pi x)$ for $\mu =1$, $\eta _{10}=\eta _2=\epsilon =0.1$, $\lambda =0.5$, and $N=10$

**Fig. 6:**
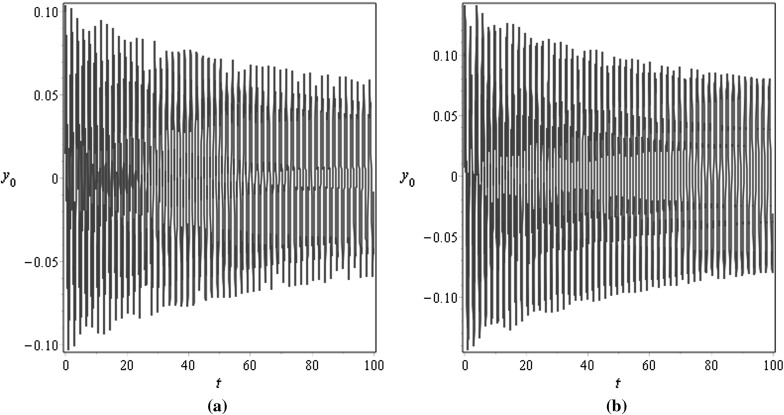
Transverse displacements $y_0$ at **a** $x=0.5$ and at **b** $x=1$ against time *t* with the initial displacement $v_0(x)=0.1\mathrm {sin}(\pi x)$ and the initial velocity $v_1(x)=0.05 \mathrm {sin}(\pi x)$ for $\mu =1$, $\eta _{10}=\eta _2=\epsilon =0.1$, $\lambda =0.05$, and $N=10$

For $\mu =0.001$, Figs. 3 and 4 demonstrate the vibration response at the middle $x=0.5$ and at the end $x=1$ points of the beam for different values of the damping factor $\tilde{\lambda }$, Fig. 3 is plotted for $\tilde{\lambda }=0.5$ and Fig. 4 is plotted for $\tilde{\lambda }=2$; the initial conditions are specified as $v_0(x)=0.1\mathrm {sin}(\pi x)$ and $v_1(x)=0.05 \mathrm {sin}(\pi x)$. For $\mu =1$, Figs. 5 and 6 illustrate similar behaviour. These figures show that the amplitudes of the transverse vibrations decrease faster for increasing of $\mu$ and $\tilde{\lambda }$. Similar results for the string-like problem have been observed in [9].

**Table 3:** Numerical approximations of $\tilde{\omega }_{n}$ in the non-resonance case for $\eta _{10}=\eta _2=\gamma _{1}=0.1$, $\sigma =2.8$, and different values of $\mu$

${\omega }_{n}$	$\tilde{\omega }_{j}$	$\tilde{\lambda }=0$	$\tilde{\lambda }=0.05$	$\tilde{\lambda }=0.1$	$\tilde{\lambda }=0.5$
$$\mu =0.001$$					
${\omega }_{1}$	$\tilde{\omega }_{1}$	0.05000 − 0.05043i	0.01553 $+$ 0.05043i	$-$ 0.01893 − 0.05043i	$-$ 0.29467 $+$ 0.05043i
	$\tilde{\omega }_{2}$	0.05000 $+$ 0.05043i	0.01553 − 0.05043i	$-$ 0.01893 $+$ 0.05043i	$-$ 0.29467 − 0.05043i
${\omega }_{2}$	$\tilde{\omega }_{1}$	0.05000 $+$ 0.12096i	0.00138 $+$ 0.12096i	$-$ 0.04724 − 0.12096i	$-$ 0.43620 $+$ 0.12096i
	$\tilde{\omega }_{2}$	0.05000 − 0.12096i	0.00138 − 0.12096i	$-$ 0.04724 $+$ 0.12096i	$-$ 0.43620 − 0.12096i
${\omega }_{3}$	$\tilde{\omega }_{1}$	0.05000 $+$ 0.19285i	$-$ 0.00293 $+$ 0.19285i	$-$ 0.05586 $+$ 0.19285i	$-$ 0.47929 $+$ 0.19285i
	$\tilde{\omega }_{2}$	0.05000 − 0.19285i	$-$ 0.00293 − 0.19285i	$-$ 0.05586 − 0.19285i	$-$ 0.47929 − 0.19285i
${\omega }_{4}$	$\tilde{\omega }_{1}$	0.05000 − 0.26094i	$-$ 0.00648 $+$ 0.26094i	$-$ 0.06295 − 0.26094i	$-$ 0.51477 − 0.26094i
	$\tilde{\omega }_{2}$	0.05000 $+$ 0.26094i	$-$ 0.00648 − 0.26094i	$-$ 0.06295 $+$ 0.26094i	$-$ 0.51477 $+$ 0.26094i
$$\mu =0.01$$					
${\omega }_{1}$	$\tilde{\omega }_{1}$	0.05000 − 0.04832i	0.01277 $+$ 0.04832i	$-$ 0.02445 − 0.04832i	$-$ 0.32227 − 0.04832i
	$\tilde{\omega }_{2}$	0.05000 $+$ 0.04832i	0.01277 − 0.04832i	$-$ 0.02445 $+$ 0.04832i	$-$ 0.32227 $+$ 0.04832i
${\omega }_{2}$	$\tilde{\omega }_{1}$	0.05000 − 0.10884i	$-$ 0.01009 $+$ 0.10884i	$-$ 0.07018 − 0.10884i	$-$ 0.55092 − 0.10884i
	$\tilde{\omega }_{2}$	0.05000 $+$ 0.10884i	$-$ 0.01009 − 0.10884i	$-$ 0.07018 $+$ 0.10884i	$-$ 0.55092 $+$ 0.10884i
${\omega }_{3}$	$\tilde{\omega }_{1}$	0.05000 $+$ 0.15541i	$-$ 0.02986 $+$ 0.15541i	$-$ 0.10972 − 0.15541i	$-$ 0.74859 − 0.15541i
	$\tilde{\omega }_{2}$	0.05000 − 0.15541i	$-$ 0.02986 − 0.15541i	$-$ 0.10972 $+$ 0.15541i	$-$ 0.74859 $+$ 0.15541i
${\omega }_{4}$	$\tilde{\omega }_{1}$	0.05000 $+$ 0.18567i	$-$ 0.05762 $+$ 0.18567i	$-$ 0.16524 $+$ 0.18567i	$-$ 1.02618 $+$ 0.18567i
	$\tilde{\omega }_{2}$	0.05000 − 0.18567i	$-$ 0.05762 − 0.18567i	$-$ 0.16524 − 0.18567i	$-$ 1.02618 − 0.18567i
$$\mu =0.1$$					
${\omega }_{1}$	$\tilde{\omega }_{1}$	0.05000 $+$ 0.04081i	0.00075 $+$ 0.04081i	$-$ 0.04851 $+$ 0.04081i	$-$ 0.44254 $+$ 0.04081i
	$\tilde{\omega }_{2}$	0.05000 − 0.04081i	0.00075 − 0.04081i	$-$ 0.04851 − 0.04081i	$-$ 0.44254 − 0.04081i
${\omega }_{2}$	$\tilde{\omega }_{1}$	0.05000 $+$ 0.06758i	$-$ 0.06988 $+$ 0.06758i	$-$ 0.18975 − 0.06758i	$-$ 1.14876 $+$ 0.06758i
	$\tilde{\omega }_{2}$	0.05000 − 0.06758i	$-$ 0.06988 − 0.06758i	$-$ 0.18975 $+$ 0.06758i	$-$ 1.14876 − 0.06758i
${\omega }_{3}$	$\tilde{\omega }_{1}$	0.05000 $+$ 0.07432i	$-$ 0.19296 $+$ 0.07432i	$-$ 0.43592 − 0.07432i	$-$ 2.37961 $+$ 0.07432i
	$\tilde{\omega }_{2}$	0.05000 − 0.07432i	$-$ 0.19296 − 0.07432i	$-$ 0.43592 $+$ 0.07432i	$-$ 2.37961 − 0.07432i
${\omega }_{4}$	$\tilde{\omega }_{1}$	0.05000 − 0.07654i	$-$ 0.37789 $+$ 0.07654i	$-$ 0.80578 − 0.07654i	$-$ 4.22886 $+$ 0.07654i
	$\tilde{\omega }_{2}$	0.05000 $+$ 0.07654i	$-$ 0.37789 − 0.07654i	$-$ 0.80578 $+$ 0.07654i	$-$ 4.22886 − 0.07654i
$$\mu =1$$					
${\omega }_{1}$	$\tilde{\omega }_{1}$	0.05000 $+$ 0.02687i	$-$ 0.01575 $+$ 0.02687i	$-$ 0.08150 $+$ 0.02687i	$-$ 0.60751 $+$ 0.02687i
	$\tilde{\omega }_{2}$	0.05000 − 0.02687i	$-$ 0.01575 − 0.02687i	$-$ 0.08150 − 0.02687i	$-$ 0.60751 − 0.02687i
${\omega }_{2}$	$\tilde{\omega }_{1}$	0.05000 $+$ 0.02550i	$-$ 0.15874 $+$ 0.02550i	$-$ 0.36747 $+$ 0.02550i	$-$ 2.03737 $+$ 0.02550i
	$\tilde{\omega }_{2}$	0.05000 − 0.02550i	$-$ 0.15874 $+$ 0.02550i	$-$ 0.36747 − 0.02550i	$-$ 2.03737 − 0.02550i
${\omega }_{3}$	$\tilde{\omega }_{1}$	0.05000 $+$ 0.02519i	$-$ 0.44062 $+$ 0.02519i	$-$ 0.93125 $+$ 0.02519i	$-$ 4.85625 $+$ 0.02519i
	$\tilde{\omega }_{2}$	0.05000 − 0.02519i	$-$ 0.44062 $+$ 0.02519i	$-$ 0.93125 − 0.02519i	$-$ 4.85625 − 0.02519i
${\omega }_{4}$	$\tilde{\omega }_{1}$	0.05000 $+$ 0.02510i	$-$ 0.86360 $+$ 0.02510i	$-$ 1.77718 $+$ 0.02510i	$-$ 9.08590 $+$ 0.02510i
	$\tilde{\omega }_{2}$	0.05000 − 0.02510i	$-$ 0.86360 $+$ 0.02510i	$-$ 1.77718 − 0.02510i	$-$ 9.08590 − 0.02510i

### The sum-type resonance case: $\varOmega _{1} = \sqrt{\omega _{2}}+ \sqrt{\omega _{1}}$

We will consider now only the first two modes and omit the higher-order modes. That is, we will assume that for the given $\mu$ values only these two modes might occur in a resonance interaction. Equation (115) can be rewritten as

124 $$\begin{aligned}&\Big [V_{1_{tt}}(t,\tau ) +\omega _{1} V_{1}(t,\tau )\Big ]= \mathrm {sin}(\sqrt{\omega _{1}}t) \Big \{ 2\sqrt{\omega _{1}}\frac{\mathrm {d} A_{1}(\tau )}{\mathrm {d} \tau }\nonumber \\&\quad +B_{1}(\tau ) \Big [\frac{\eta _{2}}{\zeta _{1}}(\hat{\varPhi }_{11}-{\varPhi _{11}}) \Big ] \nonumber \\&\quad -A_{1}(\tau ) \Big [\eta _{10}\sqrt{\omega _{1}}+\tilde{\lambda }\phi _{1}(1)\sqrt{\omega _{1}}(c_{1}\omega _{1}+d_{1}) \Big ] \Big \}\nonumber \\&\quad +\mathrm {cos}(\sqrt{\omega _{1}}t) \Big \{-2\sqrt{\omega _{1}}\frac{\mathrm {d} B_{1}(\tau )}{\mathrm {d} \tau }\nonumber \\&\quad +A_{1}(\tau ) \Big [\frac{\eta _{2}}{\zeta _{1}}(\hat{\varPhi }_{11}-{\varPhi _{11}}) \Big ] \nonumber \\&\quad +B_{1}(\tau ) \Big [\eta _{10}\sqrt{\omega _{1}}+\tilde{\lambda }\phi _{1}(1)\sqrt{\omega _{1}}(c_{1}\omega _{1}+d_{1}) \Big ] \Big \} \nonumber \\&\quad +\mathrm {sin}(\varOmega _{1}t-\sqrt{\omega _{2}}t) \frac{\sigma }{2\zeta {1}} (\varOmega _{1}\sqrt{\omega _{2}}-\omega _{2})\times \nonumber \\&\quad \Big [A_{2}(\tau ) {\varPsi }_{21}+B_{2}(\tau ){\hat{\varPsi }_{21}} \Big ] \nonumber \\&\quad +\mathrm {cos}(\varOmega _{1}t-\sqrt{\omega _{2}}t) \frac{\sigma }{2\zeta {1}} (\varOmega _{1}\sqrt{\omega _{2}}-\omega _{2})\times \nonumber \\&\quad \Big [-A_{2}(\tau ) \hat{\varPsi }_{21}+B_{2}(\tau ){{\varPsi }_{21}} \Big ]\nonumber \\&\quad +``\text {NST}'', \end{aligned}$$

and a similar equation for $V_{2}(t,\tau )$ can be obtained from Eq. (124). In order to avoid secular terms, it follows from the equations for $V_{1}(t,\tau )$ and $V_{2}(t,\tau )$ that $A_{1}(\tau )$, $B_{1}(\tau )$, $A_{2}(\tau )$ and $B_{2}(\tau )$ have to satisfy

125 $$\begin{aligned}&\frac{\mathrm {d} A_{1}(\tau )}{\mathrm {d} \tau }=A_{1}(\tau ) X_{1}-B_{1}(\tau ) Y_{1}-A_{2}(\tau ) Z_{2}\nonumber \\&\qquad \qquad \qquad -B_{2}(\tau ) C_{2}, \end{aligned}$$

126 $$\begin{aligned}&\frac{\mathrm {d} B_{1}(\tau )}{\mathrm {d} \tau }=A_{1}(\tau ) Y_{1}+B_{1}(\tau ) X_{1}-A_{2}(\tau ) C_{2}\nonumber \\&\quad +B_{2}(\tau ) Z_{2}, \end{aligned}$$

127 $$\begin{aligned}&\frac{\mathrm {d} A_{2}(\tau )}{\mathrm {d} \tau }=-A_{1}(\tau ) Z_{1}-B_{1}(\tau ) C_{1}+A_{2}(\tau ) X_{2}\nonumber \\&\quad -B_{2}(\tau ) Y_{2}, \end{aligned}$$

128 $$\begin{aligned}&\frac{\mathrm {d} B_{2}(\tau )}{\mathrm {d} \tau }=-A_{1}(\tau ) C_{1}+B_{1}(\tau ) Z_{1}+A_{2}(\tau ) Y_{2}\nonumber \\&\quad +B_{2}(\tau ) X_{2}, \end{aligned}$$

**Table 4:** Numerical approximations of $\tilde{\omega }_{n}$ in the sum-type resonance case for $\eta _{10}=\eta _2=\gamma _{1}=0.1$, $\sigma =2.8$, and different values of $\mu$

$\tilde{\omega }$	$\tilde{\lambda }=0$	$\tilde{\lambda }=0.05$	$\tilde{\lambda }=0.1$	$\tilde{\lambda }=0.5$
$$\mu =0.001$$				
$\tilde{\omega }_{1}$	0.04845 $+$ 0.11079i	0.01800 $+$ 0.04043i	$-$ 0.05054 $+$ 0.11145i	$-$ 0.44189 $+$ 0.11611i
$\tilde{\omega }_{2}$	0.04845 − 0.11079i	0.01800 − 0.04043i	$-$ 0.01564 $+$ 0.04085i	$-$ 0.44189 − 0.11611i
$\tilde{\omega }_{3}$	0.05155 $+$ 0.04017i	$-$ 0.00109 $+$ 0.11104i	$-$ 0.01564 − 0.04085i	$-$ 0.28898 $+$ 0.04557i
$\tilde{\omega }_{4}$	0.05155 − 0.04017i	$-$ 0.00109 − 0.11104i	$-$ 0.05054 − 0.11145i	$-$ 0.28898 − 0.04557i
$$\mu =0.01$$				
$\tilde{\omega }_{1}$	0.04786 $+$ 0.09626i	0.01689 $+$ 0.03638i	$-$ 0.07576 $+$ 0.09834i	$-$ 0.31627 $+$ 0.04557i
$\tilde{\omega }_{2}$	0.04786 − 0.09626i	0.01689 − 0.03638i	$-$ 0.07576 − 0.09834i	$-$ 0.31627 − 0.04557i
$\tilde{\omega }_{3}$	0.05214 $+$ 0.03554i	$-$ 0.01421 $+$ 0.09705i	$-$ 0.01887 $+$ 0.03772i	$-$ 0.55691 $+$ 0.10610i
$\tilde{\omega }_{4}$	0.05214 − 0.03554i	$-$ 0.01421 − 0.09705i	$-$ 0.01887 − 0.03772i	$-$ 0.55691 − 0.10610i
$$\mu =0.1$$				
$\tilde{\omega }_{1}$	0.03514 $+$ 0.02827i	0.01955 $+$ 0.02646i	$-$ 0.03344 $+$ 0.03425i	$-$ 0.43828 $+$ 0.04090i
$\tilde{\omega }_{2}$	0.03514 − 0.02827i	0.01955 − 0.02646i	$-$ 0.03344 − 0.03425i	$-$ 0.43828 − 0.04090i
$\tilde{\omega }_{3}$	0.07761 $+$ 0i	$-$ 0.08868 $+$ 0.05346i	$-$ 0.20482 $+$ 0.06108i	$-$ 1.15302 $+$ 0.06767i
$\tilde{\omega }_{4}$	0.05210 − 0i	$-$ 0.08868 − 0.05346i	$-$ 0.20482 − 0.06108i	$-$ 1.15302 − 0.06767i
$$\mu =1$$				
$\tilde{\omega }_{1}$	0.15268 $+$ 0.00330i	0.03971 $+$ 0.02213i	$-$ 0.04650 $+$ 0.02694i	$-$ 0.59965 $+$ 0.02800i
$\tilde{\omega }_{2}$	0.15268 − 0.00330i	0.03971 − 0.02213i	$-$ 0.04650 − 0.02694i	$-$ 0.59965 − 0.02800i
$\tilde{\omega }_{3}$	$-$ 0.04855 − 0i	$-$ 0.21419 $+$ 0.02066i	$-$ 0.40248 $+$ 0.02553i	$-$ 2.04522 $+$ 0.02662i
$\tilde{\omega }_{4}$	$-$ 0.05682 − 0i	$-$ 0.21419 − 0.02066i	$-$ 0.40248 − 0.02553i	$-$ 2.04522 − 0.02662i

**Table 5:** Numerical approximations of $\tilde{\omega }_{n}$ in the difference-type resonance case for $\eta _{10}=\eta _2=\gamma _{1}=0.1$, $\sigma =2.8$, and different values of $\mu$

$\tilde{\omega }$	$\tilde{\lambda }=0$	$\tilde{\lambda }=0.05$	$\tilde{\lambda }=0.1$	$\tilde{\lambda }=0.5$
$$\mu =0.001$$				
$\tilde{\omega }_{1}$	0.02801 $+$ 0.09637i	0.03561 $+$ 0.06786i	$-$ 0.06584 $+$ 0.10811i	$-$ 0.44619 $+$ 0.11951i
$\tilde{\omega }_{2}$	0.02801 − 0.09637i	0.03561 − 0.06786i	$-$ 0.06584 − 0.10811i	$-$ 0.44619 − 0.11951i
$\tilde{\omega }_{3}$	0.07199 $+$ 0.07502i	$-$ 0.01870 $+$ 0.10353i	$-$ 0.00034 $+$ 0.06328i	$-$ 0.28469 $+$ 0.05188i
$\tilde{\omega }_{4}$	0.07199 − 0.07502i	$-$ 0.01870 − 0.10353i	$-$ 0.00034 − 0.06328i	$-$ 0.28469 − 0.05188i
$$\mu =0.01$$				
$\tilde{\omega }_{1}$	0.01910 $+$ 0.08773i	0.03746 $+$ 0.06118i	$-$ 0.09085 $+$ 0.10097i	$-$ 0.31460 $+$ 0.04791i
$\tilde{\omega }_{2}$	0.01910 − 0.08773i	0.03746 − 0.06118i	$-$ 0.09085 − 0.10097i	$-$ 0.31460 − 0.04791i
$\tilde{\omega }_{3}$	0.08090 $+$ 0.06943i	$-$ 0.03478 $+$ 0.09599i	$-$ 0.00378 $+$ 0.05619i	$-$ 0.55859 $+$ 0.10926i
$\tilde{\omega }_{4}$	0.08090 − 0.06943i	$-$ 0.03478 − 0.09599i	$-$ 0.00378 − 0.05619i	$-$ 0.55859 − 0.10926i
$$\mu =0.1$$				
$\tilde{\omega }_{1}$	0.10334 $+$ 0.04419i	0.03048 $+$ 0.03872i	$-$ 0.02966 $+$ 0.03766i	$-$ 0.43837 $+$ 0.03947i
$\tilde{\omega }_{2}$	0.10334 − 0.04419i	0.03048 − 0.03872i	$-$ 0.02966 − 0.03766i	$-$ 0.43837 − 0.03947i
$\tilde{\omega }_{3}$	$-$ 0.00334 $+$ 0.06420i	$-$ 0.09961 $+$ 0.06966i	$-$ 0.20860 $+$ 0.07072i	$-$ 1.15293 $+$ 0.06891i
$\tilde{\omega }_{4}$	$-$ 0.00334 − 0.06420i	$-$ 0.09961 −0.06966i	$-$ 0.20860 − 0.07072i	$-$ 1.15293 − 0.06891i
$$\mu =1$$				
$\tilde{\omega }_{1}$	0.15598 $+$ 0.00647i	0.04008 $+$ 0.01016i	$-$ 0.04724 $+$ 0.01495i	$-$ 0.59996 − 0.02397i
$\tilde{\omega }_{2}$	0.15598 − 0.00647i	0.04008 − 0.01016i	$-$ 0.04724 − 0.01495i	$-$ 0.59996 $+$ 0.02397i
$\tilde{\omega }_{3}$	$-$ 0.05598 $+$ 0.04590i	$-$ 0.21457 $+$ 0.04221i	$-$ 0.40173 $+$ 0.03742i	$-$ 2.04492 − 0.02840i
$\tilde{\omega }_{4}$	$-$ 0.05598 − 0.04590i	$-$ 0.21457 − 0.04221i	$-$ 0.40173 − 0.03742i	$-$ 2.04492 $+$ 0.02840i

where $X_{1}$, $X_{2}$, $Y_{1}$, $Y_{2}$, $Z_{1}$, $Z_{2}$, $C_{1}$ and $C_{2}$ are defined by

$$\begin{aligned} X_{1}= & {} \Big [ \frac{\eta _{10}}{2}+\frac{\tilde{\lambda }}{2}\phi _{1}(1)(c_{1}\omega _{1}+d_{1}) \Big ], \\ X_{2}= & {} \Big [ \frac{\eta _{10}}{2}+\frac{\tilde{\lambda }}{2}\phi _{2}(1)(c_{2}\omega _{2}+d_{2}) \Big ], \\ Y_{1}= & {} \Big [ \frac{\eta _{2}}{2\zeta _{1}\sqrt{\omega _{1}}}(\hat{\varPhi }_{11}-{\varPhi _{11}}) \Big ], \\ Y_{2}= & {} \Big [ \frac{\eta _{2}}{2\zeta _{2}\sqrt{\omega _{2}}}(\hat{\varPhi }_{22}-{\varPhi _{22}}) \Big ], \\ Z_{1}= & {} \frac{\sigma {\varPsi }_{12} }{4\zeta _{2} \sqrt{\omega _{2}}} (\varOmega _{1}\sqrt{\omega _{1}}-\omega _{1}), \\ Z_{2}= & {} \frac{\sigma {\varPsi }_{21} }{4\zeta _{1} \sqrt{\omega _{1}}} (\varOmega _{1}\sqrt{\omega _{2}}-\omega _{2}), \\ C_{1}= & {} \frac{\sigma {\hat{\varPsi }_{12}} }{4\zeta _{2} \sqrt{\omega _{2}}} (\varOmega _{1}\sqrt{\omega _{1}}-\omega _{1}), \\ C_{2}= & {} \frac{\sigma {\hat{\varPsi }_{21}} }{4\zeta _{1} \sqrt{\omega _{1}}} (\varOmega _{1}\sqrt{\omega _{2}}-\omega _{2}). \end{aligned}$$

We obtain from the system Eqs. (125)–(128),

$$\begin{aligned} \dot{X}=A X \end{aligned}$$

where

$$\begin{aligned} X=\begin{pmatrix} A_{1}(\tau ) \\ B_{1}(\tau )\\ A_{2}(\tau )\\ B_{2}(\tau )\\ \end{pmatrix},\text {and}~~~~~~A=\begin{pmatrix} X_{1} &{}-Y_{1} &{}-Z_{2} &{}-C_{2}\\ Y_{1} &{} X_{1} &{}-C_{2} &{} Z_{2}\\ -Z_{1} &{}-C_{1} &{} X_{2} &{}-Y_{2}\\ -C_{1} &{} Z_{1} &{} Y_{2} &{} X_{2}\\ \end{pmatrix}. \end{aligned}$$

and where $\dot{X}$ represents the derivative of *X* with respect to $\tau$. The matrix *A* for a given configuration is only depending on the damping parameter $\tilde{\lambda }$. The other parameters are determined by the physics, which are taken from [1, 3] in Table 4. In order to stabilise the system, we should determine the damping parameter $\tilde{\lambda }$ in such a way that all the real parts of the eigenvalues of the matrix *A* are negative. As can be seen in Table 4, if we assume $\tilde{\lambda }=0$, there is an instability in the system due to the wind force $\eta _{10}$. While for increasing values of $\tilde{\lambda }$, we observe that the cable system becomes stable, which also depends on the value of the bending stiffness $\mu$.

### The difference-type resonance case: $\varOmega _{1} = \sqrt{\omega _{2}}- \sqrt{\omega _{1}}$

A similar analysis, as given for the sum-type resonance case, can also be applied in the difference-type resonance case. We will consider $\varOmega _{1} = \sqrt{\omega _{2}}- \sqrt{\omega _{1}}$, which is assumed to have only solution $m=2$, $n=1$ (or $m=1$, $n=2$). Then, Eq. (115) can be rewritten as

129 $$\begin{aligned}&\Big [V_{1_{tt}}(t,\tau ) +\omega _{1} V_{1}(t,\tau )\Big ]= \mathrm {sin}(\sqrt{\omega _{1}}t) \Big \{ 2\sqrt{\omega _{1}}\frac{\mathrm {d} A_{1}(\tau )}{\mathrm {d} \tau }\nonumber \\&\quad +B_{1}(\tau ) \Big [\frac{\eta _{2}}{\zeta _{1}}(\hat{\varPhi }_{11}-{\varPhi _{11}}) \Big ] \nonumber \\&\quad -A_{1}(\tau ) \Big [\eta _{10}\sqrt{\omega _{1}}+\tilde{\lambda }\phi _{1}(1)\sqrt{\omega _{1}}(c_{1}\omega _{1}+d_{1}) \Big ] \Big \}\nonumber \\&\quad +\mathrm {cos}(\sqrt{\omega _{1}}t) \Big \{-2\sqrt{\omega _{1}}\frac{\mathrm {d} B_{1}(\tau )}{\mathrm {d} \tau }\nonumber \\&\quad +A_{1}(\tau ) \Big [\frac{\eta _{2}}{\zeta _{1}}(\hat{\varPhi }_{11}-{\varPhi _{11}}) \Big ] \nonumber \\&\quad +B_{1}(\tau ) \Big [\eta _{10}\sqrt{\omega _{1}}+\tilde{\lambda }\phi _{1}(1)\sqrt{\omega _{1}}(c_{1}\omega _{1}+d_{1}) \Big ] \Big \} \nonumber \\&\quad +\mathrm {sin}(\varOmega _{1}t+\sqrt{\omega _{2}}t) \frac{\sigma }{2\zeta {1}} (\varOmega _{1}\sqrt{\omega _{2}}+\omega _{2})\times \nonumber \\&\quad \Big [-A_{2}(\tau ) {\varPsi }_{21}+B_{2}(\tau ){\hat{\varPsi }_{21}} \Big ] \nonumber \\&\quad +\mathrm {cos}(\varOmega _{1}t+\sqrt{\omega _{2}}t) \frac{\sigma }{2\zeta {1}} (\varOmega _{1}\sqrt{\omega _{2}}+\omega _{2})\times \nonumber \\&\quad \Big [A_{2}(\tau ) \hat{\varPsi }_{21}+B_{2}(\tau ){{\varPsi }_{21}} \Big ] \nonumber \\&\quad +``\text {NST}'', \end{aligned}$$

and a similar equation for $V_{2}(t,\tau )$ can be obtained from Eq. (129). In order to avoid secular terms, it follows from the equations for $V_{1}(t,\tau )$ and $V_{2}(t,\tau )$ that $A_{1}(\tau )$, $B_{1}(\tau )$, $A_{2}(\tau )$ and $B_{2}(\tau )$ have to satisfy

130 $$\begin{aligned} \frac{\mathrm {d} A_{1}(\tau )}{\mathrm {d} \tau }= & {} A_{1}(\tau ) X_{1}-B_{1}(\tau ) Y_{1}+A_{2}(\tau ) \tilde{Z}_{2} \nonumber \\&\quad -B_{2}(\tau ) \tilde{C}_{2}, \end{aligned}$$

131 $$\begin{aligned} \frac{\mathrm {d} B_{1}(\tau )}{\mathrm {d} \tau }= & {} A_{1}(\tau ) Y_{1}+B_{1}(\tau ) X_{1}+A_{2}(\tau ) \tilde{C}_{2}\nonumber \\&\quad +B_{2}(\tau ) \tilde{Z}_{2}, \end{aligned}$$

132 $$\begin{aligned} \frac{\mathrm {d} A_{2}(\tau )}{\mathrm {d} \tau }= & {} A_{1}(\tau ) \tilde{Z}_{1}-B_{1}(\tau ) \tilde{C}_{1}\nonumber \\&\quad +A_{2}(\tau ) X_{2}-B_{2}(\tau ) Y_{2}, \end{aligned}$$

133 $$\begin{aligned} \frac{\mathrm {d} B_{2}(\tau )}{\mathrm {d} \tau }= & {} A_{1}(\tau ) \tilde{C}_{1}+B_{1}(\tau ) \tilde{Z}_{1}\nonumber \\&\quad +A_{2}(\tau ) Y_{2}+B_{2}(\tau ) X_{2}, \end{aligned}$$

where $X_{1}$, $X_{2}$, $Y_{1}$, $Y_{2}$, $\tilde{Z}_{1}$, $\tilde{Z}_{2}$, $\tilde{C}_{1}$ and $\tilde{C}_{2}$ are defined by:

$$\begin{aligned} X_{1}= & {} \Big [ \frac{\eta _{10}}{2}+\frac{\tilde{\lambda }}{2}\phi _{1}(1)(c_{1}\omega _{1}+d_{1}) \Big ], \\ X_{2}= & {} \Big [ \frac{\eta _{10}}{2}+\frac{\tilde{\lambda }}{2}\phi _{2}(1)(c_{2}\omega _{2}+d_{2}) \Big ], \\ Y_{1}= & {} \Big [ \frac{\eta _{2}}{2\zeta _{1}\sqrt{\omega _{1}}}(\hat{\varPhi }_{11}-{\varPhi _{11}}) \Big ], \\ Y_{2}= & {} \Big [ \frac{\eta _{2}}{2\zeta _{2}\sqrt{\omega _{2}}}(\hat{\varPhi }_{22}-{\varPhi _{22}}) \Big ], \\ \tilde{Z}_{1}= & {} \frac{\sigma {\varPsi }_{12} }{4\zeta _{2} \sqrt{\omega _{2}}} (\varOmega _{1}\sqrt{\omega _{1}}+\omega _{1}), \\ \tilde{Z}_{2}= & {} \frac{\sigma {\varPsi }_{21} }{4\zeta _{1} \sqrt{\omega _{1}}} (\varOmega _{1}\sqrt{\omega _{2}}+\omega _{2}), \\ \tilde{C}_{1}= & {} \frac{\sigma {\hat{\varPsi }_{12}} }{4\zeta _{2} \sqrt{\omega _{2}}} (\varOmega _{1}\sqrt{\omega _{1}}+\omega _{1}), \\ \tilde{C}_{2}= & {} \frac{\sigma {\hat{\varPsi }_{21}} }{4\zeta _{1} \sqrt{\omega _{1}}} (\varOmega _{1}\sqrt{\omega _{2}}+\omega _{2}). \end{aligned}$$

We obtain from the system Eqs. (130)–(133),

$$\begin{aligned} \dot{X}=A X \end{aligned}$$

where

$$\begin{aligned} X=\begin{pmatrix} A_{1}(\tau ) \\ B_{1}(\tau )\\ A_{2}(\tau )\\ B_{2}(\tau )\\ \end{pmatrix},\text {and}~~~~~~A=\begin{pmatrix} X_{1} &{}-Y_{1} &{}\tilde{Z}_{2} &{}-\tilde{C}_{2}\\ Y_{1} &{} X_{1} &{}\tilde{C}_{2} &{} \tilde{Z}_{2}\\ \tilde{Z}_{1} &{}-\tilde{C}_{1} &{} X_{2} &{}-Y_{2}\\ \tilde{C}_{1} &{} \tilde{Z}_{1} &{} Y_{2} &{} X_{2}\\ \end{pmatrix}. \end{aligned}$$

and where $\dot{X}$ represents the derivative of *X* with respect to $\tau$. As in the sum-type resonance case, this matrix *A* for a given configuration is only depending on the damping parameter $\tilde{\lambda }$. In Table 5, it can easily be seen that there is a change from instability to stability, which is around $\tilde{\lambda }=0.05$.

## Conclusions

In this paper, initial-boundary value problems for a tensioned beam equation are studied. The model is derived to describe the rain–wind-induced oscillations of an inclined cable. We applied a multiple-timescales perturbation method in order to observe whether or not mode interactions between vibration modes occur for certain values of the bending stiffness and the damping parameter. The results show that the system in both the pure resonance case and the non-resonance case can be stabilised by a boundary damper. Some of these cases are studied in Sect. 4. Mode interactions between two and more modes depending on the bending stiffness $\mu$ are possible. More complicated resonance cases can be $\varOmega _{1} = \sqrt{\omega _{n}} \pm \sqrt{\omega _{m}}$ or $\varOmega _{1} = 2 \sqrt{\omega _{n}}$, when $\varOmega _{1} = \sqrt{\omega _{N}} + \sqrt{\omega _{M}}$ ( or $\varOmega _{1} = \sqrt{\omega _{N}} - \sqrt{\omega _{M}}$ ) for some fixed *N* and *M*. These cases still have to be studied, and can be studied by using the techniques as shown in Sect. 4 of this paper.

This paper provides an understanding of how effective boundary damping can be for the in-plane transversal oscillations of the cable. The same approach can be used for in-plane and out-of-plane transversal oscillations of elastic structures.

## Appendix A: Aerodynamic parameters

134 $$\begin{aligned} b&:=[ -(\psi _{1}+\alpha _{1})+ \mathrm {arctan}(\mathrm {tan}(\gamma ))], \end{aligned}$$

135 $$\begin{aligned} c&:=[ -(\psi _{2}+\alpha _{2})+ \mathrm {arctan}(\mathrm {tan}(\gamma ))], \end{aligned}$$

136 $$\begin{aligned} a_{000}&:=\mathrm {sin}(\gamma ), \end{aligned}$$

137 $$\begin{aligned} a_{001}&:={b}~ \mathrm {cos}(\gamma ), \end{aligned}$$

138 $$\begin{aligned} a_{002}&:={c}~ \mathrm {cos}(\gamma ), \end{aligned}$$

139 $$\begin{aligned} a_{003}&:={b^3} \mathrm {cos}(\gamma ), \end{aligned}$$

140 $$\begin{aligned} a_{004}&:={c^3} \mathrm {cos}(\gamma ), \end{aligned}$$

141 $$\begin{aligned} a_{100}&:=2-\mathrm {cos}^2(\gamma ), \end{aligned}$$

142 $$\begin{aligned} a_{101}&:=\mathrm {cos}(\gamma ) [\mathrm {cos}(\gamma ) -b~\mathrm {sin}(\gamma )], \end{aligned}$$

143 $$\begin{aligned} a_{102}&:=\mathrm {cos}(\gamma ) [\mathrm {cos}(\gamma ) -c~\mathrm {sin}(\gamma )], \end{aligned}$$

144 $$\begin{aligned} a_{103}&:={b^2} \mathrm {cos}(\gamma )[3\mathrm {cos}(\gamma ) -b~\mathrm {sin}(\gamma ) ], \end{aligned}$$

145 $$\begin{aligned} a_{104}&:={c^2} \mathrm {cos}(\gamma )[3\mathrm {cos}(\gamma ) -c~\mathrm {sin}(\gamma ) ], \end{aligned}$$

146 $$\begin{aligned} a_{010}&:=-\mathrm {sin}(\gamma )\mathrm {cos}(\gamma ) , \end{aligned}$$

147 $$\begin{aligned} a_{011}&:= \mathrm {sin}(\gamma )[\mathrm {cos}(\gamma )- b~\mathrm {sin}(\gamma )] , \end{aligned}$$

148 $$\begin{aligned} a_{012}&:=\mathrm {sin}(\gamma )[\mathrm {cos}(\gamma )- c~\mathrm {sin}(\gamma )] , \end{aligned}$$

149 $$\begin{aligned} a_{013}&:= {b^2} \mathrm {sin}(\gamma )[3\mathrm {cos}(\gamma ) -b~\mathrm {sin}(\gamma ) ] , \end{aligned}$$

150 $$\begin{aligned} a_{014}&:= {c^2} \mathrm {sin}(\gamma )[3\mathrm {cos}(\gamma ) -c~\mathrm {sin}(\gamma ) ] , \end{aligned}$$

151 $$\begin{aligned} a_{200}&:= \frac{\mathrm {sin}(\gamma )}{2}[2+ \mathrm {cos}^2(\gamma )] , \end{aligned}$$

152 $$\begin{aligned} a_{201}&:= \frac{\mathrm {cos}(\gamma )}{2}[2b-3b~\mathrm {cos}^2(\gamma )-2~\mathrm {sin}(2\gamma )] , \end{aligned}$$

153 $$\begin{aligned} a_{202}&:=\frac{\mathrm {cos}(\gamma )}{2}[2c-3c~\mathrm {cos}^2(\gamma )-2~\mathrm {sin}(2\gamma )] , \end{aligned}$$

154 $$\begin{aligned} a_{203}&:= \frac{{b}~\mathrm {cos}(\gamma )}{2}[2b^2+\mathrm {cos}^2(\gamma )(6-{3b^2})\nonumber \\&\quad -6b~\mathrm {sin}(2\gamma )] , \end{aligned}$$

155 $$\begin{aligned} a_{204}&:= \frac{{c}~\mathrm {cos}(\gamma )}{2}[2c^2+\mathrm {cos}^2(\gamma )(6-{3c^2})\nonumber \\&\quad -6c~\mathrm {sin}(2\gamma )] , \end{aligned}$$

156 $$\begin{aligned} a_{110}&:= \mathrm {cos}^3(\gamma ) , \end{aligned}$$

157 $$\begin{aligned} a_{111}&:= 4\mathrm {cos}^3(\gamma )-3\mathrm {cos}(\gamma ) +b[\mathrm {sin}(\gamma )\nonumber \\&\quad -3\mathrm {sin}(\gamma )\mathrm {cos}^2(\gamma )] , \end{aligned}$$

158 $$\begin{aligned} a_{112}&:= 4\mathrm {cos}^3(\gamma )-3\mathrm {cos}(\gamma ) +c[\mathrm {sin}(\gamma )\nonumber \\&\quad -3\mathrm {sin}(\gamma )\mathrm {cos}^2(\gamma )] , \end{aligned}$$

159 $$\begin{aligned} a_{113}&:= 6b\mathrm {sin}(\gamma )\mathrm {cos}^2(\gamma )+ b^2[12 \mathrm {cos}^3(\gamma )-9\mathrm {cos}(\gamma )]\nonumber \\&\quad + b^3[-3\mathrm {sin}(\gamma )\mathrm {cos}^2(\gamma )+\mathrm {sin}(\gamma )] , \end{aligned}$$

160 $$\begin{aligned} a_{114}&:= 6c\mathrm {sin}(\gamma )\mathrm {cos}^2(\gamma )+ c^2[12 \mathrm {cos}^3(\gamma )-9\mathrm {cos}(\gamma )]\nonumber \\&\quad + c^3[-3\mathrm {sin}(\gamma )\mathrm {cos}^2(\gamma )+\mathrm {sin}(\gamma )] , \end{aligned}$$

161 $$\begin{aligned} a_{020}&:= - \frac{\mathrm {sin}(\gamma )}{2}[3+\mathrm {cos}^2(\gamma )] , \end{aligned}$$

162 $$\begin{aligned} a_{021}&:= 2 \mathrm {sin}(\gamma ) \mathrm {cos}^2(\gamma )- \mathrm {sin}(\gamma )-\frac{3 b}{2}\mathrm {sin}^2(\gamma ) \mathrm {cos}(\gamma ), \end{aligned}$$

163 $$\begin{aligned} a_{022}&:= 2 \mathrm {sin}(\gamma ) \mathrm {cos}^2(\gamma )- \mathrm {sin}(\gamma )-\frac{3 c}{2}\mathrm {sin}^2(\gamma ) \mathrm {cos}(\gamma ), \end{aligned}$$

164 $$\begin{aligned} a_{023}&:= 3b~ \mathrm {sin}^2(\gamma ) \mathrm {cos}(\gamma )+ b^2[6~ \mathrm {sin}(\gamma ) \mathrm {cos}^2(\gamma )\nonumber \\&\quad -3~\mathrm {sin}(\gamma )]- \frac{3b^3}{2}\mathrm {sin}^2(\gamma ) \mathrm {cos}(\gamma ), \end{aligned}$$

165 $$\begin{aligned} a_{024}&:= 3c~ \mathrm {sin}^2(\gamma ) \mathrm {cos}(\gamma )+ c^2[6~ \mathrm {sin}(\gamma ) \mathrm {cos}^2(\gamma )\nonumber \\&\quad -3~\mathrm {sin}(\gamma )]- \frac{3c^3}{2}\mathrm {sin}^2(\gamma ) \mathrm {cos}(\gamma ), \end{aligned}$$

166 $$\begin{aligned} a_{300}&:= \frac{\mathrm {cos}^2(\gamma )}{2} [5~\mathrm {cos}^2(\gamma )-4], \end{aligned}$$

167 $$\begin{aligned} a_{301}&:= -\frac{\mathrm {cos}(\gamma )}{6}[-15b~\mathrm {sin}(\gamma ) \mathrm {cos}^2(\gamma )+23~\mathrm {cos}^3(\gamma )\nonumber \\&\quad +6b~\mathrm {sin}(\gamma )-18~\mathrm {cos}(\gamma ) ] , \end{aligned}$$

168 $$\begin{aligned} a_{302}&:= -\frac{\mathrm {cos}(\gamma )}{6}[-15c~\mathrm {sin}(\gamma ) \mathrm {cos}^2(\gamma )+23~\mathrm {cos}^3(\gamma )\nonumber \\&\quad +6c~\mathrm {sin}(\gamma )-18~\mathrm {cos}(\gamma ) ] , \end{aligned}$$

169 $$\begin{aligned} a_{303}&:= \Big ( 1-\frac{23b^2}{2} \Big ) \mathrm {cos}^4(\gamma ) +\Big ( \frac{5b^3}{2} -9b\Big ) \mathrm {sin}(\gamma )\mathrm {cos}^3(\gamma ) \nonumber \\&\quad +9b^2~\mathrm {cos}^2(\gamma )-b^3~\mathrm {sin}(\gamma )\mathrm {cos}(\gamma ), \end{aligned}$$

170 $$\begin{aligned} a_{304}&:= \Big ( 1-\frac{23c^2}{2} \Big ) \mathrm {cos}^4(\gamma ) +\Big ( \frac{5c^3}{2} -9c\Big ) \mathrm {sin}(\gamma )\mathrm {cos}^3(\gamma )\nonumber \\&\quad +9c^2~\mathrm {cos}^2(\gamma )-c^3~\mathrm {sin}(\gamma )\mathrm {cos}(\gamma ), \end{aligned}$$

171 $$\begin{aligned} a_{210}&:= \frac{3}{2}\mathrm {sin}(\gamma )\mathrm {cos}^3(\gamma ) , \end{aligned}$$

172 $$\begin{aligned} a_{211}&:= b~[\frac{15}{2}\mathrm {cos}^2(\gamma )- \frac{15}{2}\mathrm {cos}^4(\gamma )-1]\nonumber \\&\quad +5\mathrm {sin}(\gamma )\mathrm {cos}(\gamma )-\frac{23}{2}\mathrm {sin}(\gamma )\mathrm {cos}^3(\gamma ) , \end{aligned}$$

173 $$\begin{aligned} a_{212}&:= c~[\frac{15}{2}\mathrm {cos}^2(\gamma )- \frac{15}{2}\mathrm {cos}^4(\gamma )-1]\nonumber \\&\quad +5\mathrm {sin}(\gamma )\mathrm {cos}(\gamma )-\frac{23}{2}\mathrm {sin}(\gamma )\mathrm {cos}^3(\gamma ) , \end{aligned}$$

174 $$\begin{aligned} a_{213}&:=\Big (27b-\frac{15b^3}{2}\Big )\mathrm {cos}^4(\gamma )\nonumber \\&\quad +\Big (3-\frac{69b^2}{2}\Big )\mathrm {sin}(\gamma ) \mathrm {cos}^3(\gamma )\nonumber \\&\quad +\Big (\frac{15b^3}{2}-21b\Big )\mathrm {cos}^2(\gamma )\nonumber \\&\quad +15b^2 \mathrm {sin}(\gamma ) \mathrm {cos}(\gamma )-b^3, \end{aligned}$$

175 $$\begin{aligned} a_{214}&:= \Big (27c-\frac{15c^3}{2}\Big )\mathrm {cos}^4(\gamma )\nonumber \\&\quad +\Big (3-\frac{69c^2}{2}\Big )\mathrm {sin}(\gamma ) \mathrm {cos}^3(\gamma )\nonumber \\&\quad +\Big (\frac{15c^3}{2}-21c\Big )\mathrm {cos}^2(\gamma )\nonumber \\&\quad +15c^2 \mathrm {sin}(\gamma ) \mathrm {cos}(\gamma )-c^3 , \end{aligned}$$

176 $$\begin{aligned} a_{120}&:= \frac{3}{2}\mathrm {sin}^2(\gamma )\mathrm {cos}^2(\gamma ), \end{aligned}$$

177 $$\begin{aligned} a_{121}&:=b \Big (-\frac{15}{2}\mathrm {sin}(\gamma )\mathrm {cos}^3(\gamma )+ \frac{9}{2}\mathrm {sin}(\gamma )\mathrm {cos}(\gamma ) \Big )\nonumber \\&\quad +\frac{17}{2}\mathrm {cos}^4(\gamma )- \frac{19}{2}\mathrm {cos}^2(\gamma )+2 , \end{aligned}$$

178 $$\begin{aligned} a_{122}&:=c \Big (-\frac{15}{2}\mathrm {sin}(\gamma )\mathrm {cos}^3(\gamma )+ \frac{9}{2}\mathrm {sin}(\gamma )\mathrm {cos}(\gamma ) \Big )\nonumber \\&\quad +\frac{17}{2}\mathrm {cos}^4(\gamma )- \frac{19}{2}\mathrm {cos}^2(\gamma )+2, \end{aligned}$$

179 $$\begin{aligned} a_{123}&:= \Big (\frac{51b^2}{2}-3\Big )\mathrm {cos}^4(\gamma )\nonumber \\&\quad +\Big (27b-\frac{15b^3}{2}\Big )\mathrm {sin}(\gamma ) \mathrm {cos}^3(\gamma )\nonumber \\&\quad +\Big (3-\frac{57b^2}{2}\Big )\mathrm {cos}^2(\gamma )\nonumber \\&\quad +\Big (\frac{9b^3}{2}- 15b\Big ) \mathrm {sin}(\gamma ) \mathrm {cos}(\gamma )+6b^2 , \end{aligned}$$

180 $$\begin{aligned} a_{124}&:= \Big (\frac{51c^2}{2}-3\Big )\mathrm {cos}^4(\gamma )\nonumber \\&\quad +\Big (27c-\frac{15c^3}{2}\Big )\mathrm {sin}(\gamma ) \mathrm {cos}^3(\gamma )\nonumber \\&\quad +\Big (3-\frac{57c^2}{2}\Big )\mathrm {cos}^2(\gamma )\nonumber \\&\quad +\Big (\frac{9c^3}{2}- 15c\Big ) \mathrm {sin}(\gamma ) \mathrm {cos}(\gamma )+6c^2 , \end{aligned}$$

181 $$\begin{aligned} a_{030}&:= \frac{1}{2}\mathrm {sin}^3(\gamma )\mathrm {cos}(\gamma ) , \end{aligned}$$

182 $$\begin{aligned} a_{031}&:= \frac{\mathrm {sin}(\gamma )}{6}[23~\mathrm {cos}^3(\gamma )-17~\mathrm {cos}(\gamma )]\nonumber \\&\quad +\frac{b~\mathrm {sin}^2(\gamma )}{2}[1-5~\mathrm {cos}^2(\gamma )], \end{aligned}$$

183 $$\begin{aligned} a_{032}&:=\frac{\mathrm {sin}(\gamma )}{6}[23~\mathrm {cos}^3(\gamma )-17~\mathrm {cos}(\gamma )]\nonumber \\&\quad +\frac{c~\mathrm {sin}^2(\gamma )}{2}[1-5~\mathrm {cos}^2(\gamma )], \end{aligned}$$

184 $$\begin{aligned} a_{033}&:= \Big (\frac{23b^2}{2} -1\Big ) \mathrm {cos}^3(\gamma )\mathrm {sin}(\gamma )\nonumber \\&\quad -\Big ( \frac{5b^3}{2} -9b\Big ) \mathrm {sin}^2(\gamma )\mathrm {cos}^2(\gamma )\nonumber \\&\quad +\Big (1- \frac{17b^2}{2} \Big ) \mathrm {sin}(\gamma )\mathrm {cos}(\gamma )\nonumber \\&\quad +\Big (\frac{b^3}{2} -3b\Big ) \mathrm {sin}^2(\gamma ), \end{aligned}$$

185 $$\begin{aligned} a_{034}:=&\Big (\frac{23c^2}{2}-1 \Big ) \mathrm {cos}^3(\gamma )\mathrm {sin}(\gamma )\nonumber \\&\quad -\Big ( \frac{5c^3}{2} -9c\Big ) \mathrm {sin}^2(\gamma )\mathrm {cos}^2(\gamma )\nonumber \\&\quad +\Big (1- \frac{17c^2}{2} \Big ) \mathrm {sin}(\gamma )\mathrm {cos}(\gamma )\nonumber \\&\quad +\Big (\frac{c^3}{2} -3c\Big ) \mathrm {sin}^2(\gamma ), \end{aligned}$$

186 $$\begin{aligned} b_{000}&:=\mathrm {cos}(\gamma ), \end{aligned}$$

187 $$\begin{aligned} b_{001}&:={b}~ \mathrm {sin}(\gamma ), \end{aligned}$$

188 $$\begin{aligned} b_{002}&:={c}~ \mathrm {sin}(\gamma ), \end{aligned}$$

189 $$\begin{aligned} b_{003}&:={b^3} \mathrm {sin}(\gamma ), \end{aligned}$$

190 $$\begin{aligned} b_{004}&:={c^3} \mathrm {sin}(\gamma ), \end{aligned}$$

191 $$\begin{aligned} b_{100}&:= \mathrm {sin}(\gamma )\mathrm {cos}(\gamma ), \end{aligned}$$

192 $$\begin{aligned} b_{101}&:= \mathrm {cos}(\gamma )[b~\mathrm {cos}(\gamma )+\mathrm {sin}(\gamma )], \end{aligned}$$

193 $$\begin{aligned} b_{102}&:= \mathrm {cos}(\gamma )[c~\mathrm {cos}(\gamma )+\mathrm {sin}(\gamma )], \end{aligned}$$

194 $$\begin{aligned} b_{103}&:= {b^2} \mathrm {cos}(\gamma )[3\mathrm {sin}(\gamma ) +b~\mathrm {cos}(\gamma ) ], \end{aligned}$$

195 $$\begin{aligned} b_{104}&:= {c^2} \mathrm {cos}(\gamma )[3\mathrm {sin}(\gamma ) +c~\mathrm {cos}(\gamma ) ], \end{aligned}$$

196 $$\begin{aligned} b_{010}&:= -1- \mathrm {cos}^2(\gamma ), \end{aligned}$$

197 $$\begin{aligned} b_{011}&:= \mathrm {sin}(\gamma )[b~\mathrm {cos}(\gamma )+\mathrm {sin}(\gamma )], \end{aligned}$$

198 $$\begin{aligned} b_{012}&:= \mathrm {sin}(\gamma )[c~\mathrm {cos}(\gamma )+\mathrm {sin}(\gamma )], \end{aligned}$$

199 $$\begin{aligned} b_{013}&:= {b^2} \mathrm {sin}(\gamma )[3\mathrm {sin}(\gamma ) +b~\mathrm {cos}(\gamma ) ], \end{aligned}$$

200 $$\begin{aligned} b_{014}&:= {c^2} \mathrm {sin}(\gamma )[3\mathrm {sin}(\gamma ) +c~\mathrm {cos}(\gamma ) ], \end{aligned}$$

201 $$\begin{aligned} b_{200}&:= \frac{\mathrm {cos}^3(\gamma )}{2} , \end{aligned}$$

202 $$\begin{aligned} b_{201}&:= -\frac{\mathrm {cos}(\gamma )}{2}[2-4~\mathrm {cos}^2(\gamma )+3b~\mathrm {sin}(\gamma )\mathrm {cos}(\gamma )] , \end{aligned}$$

203 $$\begin{aligned} b_{202}&:= -\frac{\mathrm {cos}(\gamma )}{2}[2-4~\mathrm {cos}^2(\gamma )+3c~\mathrm {sin}(\gamma )\mathrm {cos}(\gamma )] , \end{aligned}$$

204 $$\begin{aligned} b_{203}&:= \frac{-3b}{2}\mathrm {cos}(\gamma ) [b^2~\mathrm {sin}(\gamma )\mathrm {cos}(\gamma )-4b~\mathrm {cos}^2(\gamma )\nonumber \\&\quad -2\mathrm {sin}(\gamma )\mathrm {cos}(\gamma )+2b] , \end{aligned}$$

205 $$\begin{aligned} b_{204}&:= \frac{-3c}{2}\mathrm {cos}(\gamma ) [c^2~\mathrm {sin}(\gamma )\mathrm {cos}(\gamma )-4c~\mathrm {cos}^2(\gamma )\nonumber \\&\quad -2\mathrm {sin}(\gamma )\mathrm {cos}(\gamma )+2c] , \end{aligned}$$

206 $$\begin{aligned} b_{110}&:= -\mathrm {sin}^3(\gamma ), \end{aligned}$$

207 $$\begin{aligned} b_{111}&:= b~[3\mathrm {cos}^3(\gamma )-2\mathrm {cos}(\gamma )]\nonumber \\&\quad +4\mathrm {sin}(\gamma )\mathrm {cos}^2(\gamma )-\mathrm {sin}(\gamma ) , \end{aligned}$$

208 $$\begin{aligned} b_{112}&:= c~[3\mathrm {cos}^3(\gamma )-2\mathrm {cos}(\gamma )]\nonumber \\&\quad +4\mathrm {sin}(\gamma )\mathrm {cos}^2(\gamma )-\mathrm {sin}(\gamma ) , \end{aligned}$$

209 $$\begin{aligned} b_{113}&:= b^3~[3\mathrm {cos}^3(\gamma )-2\mathrm {cos}(\gamma )]\nonumber \\&\quad +3b^2~[4\mathrm {cos}^2(\gamma )\mathrm {sin}(\gamma )-\mathrm {sin}(\gamma )]\nonumber \\&\quad -6b[\mathrm {cos}^3(\gamma )-\mathrm {cos}(\gamma )] , \end{aligned}$$

210 $$\begin{aligned} b_{114}&:= c^3~[3\mathrm {cos}^3(\gamma )-2\mathrm {cos}(\gamma )]\nonumber \\&\quad +3c^2~[4\mathrm {cos}^2(\gamma )\mathrm {sin}(\gamma )-\mathrm {sin}(\gamma )]\nonumber \\&\quad -6c[\mathrm {cos}^3(\gamma )-\mathrm {cos}(\gamma )] , \end{aligned}$$

211 $$\begin{aligned} b_{020}&:= -\frac{\mathrm {cos}(\gamma )}{2}[\mathrm {cos}^2(\gamma )-3] , \end{aligned}$$

212 $$\begin{aligned} b_{021}&:= \frac{\mathrm {sin}(\gamma )}{2}[3b~\mathrm {cos}^2(\gamma )+4\mathrm {sin}(\gamma )\mathrm {cos}(\gamma )-b] , \end{aligned}$$

213 $$\begin{aligned} b_{022}&:= \frac{\mathrm {sin}(\gamma )}{2}[3c~\mathrm {cos}^2(\gamma )+4\mathrm {sin}(\gamma )\mathrm {cos}(\gamma )-c] , \end{aligned}$$

214 $$\begin{aligned} b_{023}&:= \frac{b}{2}\mathrm {sin}(\gamma )[3b^2~\mathrm {cos}^2(\gamma ) +12b~\mathrm {sin}(\gamma )\mathrm {cos}(\gamma )\nonumber \\&\quad -6\mathrm {cos}^2(\gamma )-b^2+6] , \end{aligned}$$

215 $$\begin{aligned} b_{024}&:= \frac{c}{2}\mathrm {sin}(\gamma )[3c^2~\mathrm {cos}^2(\gamma ) +12c~\mathrm {sin}(\gamma )\mathrm {cos}(\gamma )\nonumber \\&\quad -6\mathrm {cos}^2(\gamma )-c^2+6] , \end{aligned}$$

216 $$\begin{aligned} b_{300}&:= -\frac{1}{2}\mathrm {sin}(\gamma )\mathrm {cos}^3(\gamma ) , \end{aligned}$$

217 $$\begin{aligned} b_{301}&:= -\frac{\mathrm {cos}(\gamma )}{6}[3b~\mathrm {cos}^3(\gamma )+23~\mathrm {sin}(\gamma )\mathrm {cos}^2(\gamma )\nonumber \\&\quad -6\mathrm {sin}(\gamma )] , \end{aligned}$$

218 $$\begin{aligned} b_{302}&:=-\frac{\mathrm {cos}(\gamma )}{6}[3c~\mathrm {cos}^3(\gamma )+23~\mathrm {sin}(\gamma )\mathrm {cos}^2(\gamma )\nonumber \\&\quad -6\mathrm {sin}(\gamma )] , \end{aligned}$$

219 $$\begin{aligned} b_{303}&:= \Big ( 9b-\frac{b^3}{2} \Big ) \mathrm {cos}^4(\gamma )\nonumber \\&\quad +\Big (1- \frac{23b^2}{2}\Big ) \mathrm {sin}(\gamma )\mathrm {cos}^3(\gamma )\nonumber \\&\quad -6b~\mathrm {cos}^2(\gamma ) +3b^2~\mathrm {sin}(\gamma )\mathrm {cos}(\gamma ), \end{aligned}$$

220 $$\begin{aligned} b_{304}&:= \Big ( 9c-\frac{c^3}{2} \Big ) \mathrm {cos}^4(\gamma )\nonumber \\&\quad +\Big (1- \frac{23c^2}{2}\Big ) \mathrm {sin}(\gamma )\mathrm {cos}^3(\gamma )\nonumber \\&\quad -6c~\mathrm {cos}^2(\gamma ) +3c^2~\mathrm {sin}(\gamma )\mathrm {cos}(\gamma ), \end{aligned}$$

221 $$\begin{aligned} b_{210}&:= -\frac{3}{2}\mathrm {sin}^2(\gamma )\mathrm {cos}^2(\gamma ) , \end{aligned}$$

222 $$\begin{aligned} b_{211}&:= b[-\frac{15}{2}\mathrm {cos}^3(\gamma )\mathrm {sin}(\gamma )+3\mathrm {sin}(\gamma )\mathrm {cos}(\gamma )]\nonumber \\&\quad +\frac{19}{2}\mathrm {cos}^4(\gamma )-\frac{17}{2}\mathrm {cos}^2(\gamma )+1 , \end{aligned}$$

223 $$\begin{aligned} b_{212}:=&c[-\frac{15}{2}\mathrm {cos}^3(\gamma )\mathrm {sin}(\gamma )+3\mathrm {sin}(\gamma )\mathrm {cos}(\gamma )]\nonumber \\&\quad +\frac{19}{2}\mathrm {cos}^4(\gamma )-\frac{17}{2}\mathrm {cos}^2(\gamma )+1 , \end{aligned}$$

224 $$\begin{aligned} b_{213}&:= \Big (\frac{69b^2}{2} -3 \Big ) \mathrm {cos}^4(\gamma )\nonumber \\&\quad +\Big ( 27b-\frac{15b^3}{2}\Big ) \mathrm {cos}^3(\gamma ) \mathrm {sin}(\gamma )\nonumber \\&\quad + \Big (3-\frac{63b^2}{2} \Big ) \mathrm {cos}^2(\gamma )\nonumber \\&\quad +\Big ( 3b^3-12b\Big ) \mathrm {cos}(\gamma ) \mathrm {sin}(\gamma )+3b^2 , \end{aligned}$$

225 $$\begin{aligned} b_{214}&:= \Big (\frac{69c^2}{2} -3 \Big ) \mathrm {cos}^4(\gamma )\nonumber \\&\quad +\Big ( 27c-\frac{15c^3}{2}\Big ) \mathrm {cos}^3(\gamma ) \mathrm {sin}(\gamma )\nonumber \\&\quad + \Big (3-\frac{63c^2}{2} \Big ) \mathrm {cos}^2(\gamma )\nonumber \\&\quad +\Big ( 3c^3-12c\Big ) \mathrm {cos}(\gamma ) \mathrm {sin}(\gamma )+3c^2 , \end{aligned}$$

226 $$\begin{aligned} b_{120}&:= -\frac{3}{2}\mathrm {cos}(\gamma ) \mathrm {sin}^3(\gamma ) , \end{aligned}$$

227 $$\begin{aligned} b_{121}&:= b[\frac{15}{2}\mathrm {cos}^4 (\gamma )-\frac{15}{2} \mathrm {cos}^2(\gamma )+1]\nonumber \\&\quad +\frac{23}{2}\mathrm {cos}^3(\gamma ) \mathrm {sin}(\gamma )- \frac{13}{2}\mathrm {cos}(\gamma ) \mathrm {sin}(\gamma ) , \end{aligned}$$

228 $$\begin{aligned} b_{122}&:= c[\frac{15}{2}\mathrm {cos}^4 (\gamma )-\frac{15}{2} \mathrm {cos}^2(\gamma )+1]\nonumber \\&\quad +\frac{23}{2}\mathrm {cos}^3(\gamma ) \mathrm {sin}(\gamma )\nonumber \\&\quad -\frac{13}{2}\mathrm {cos}(\gamma ) \mathrm {sin}(\gamma ) , \end{aligned}$$

229 $$\begin{aligned} b_{123}&:= \Big ( \frac{15b^3}{2}-27b\Big )\mathrm {cos}^4 (\gamma )\nonumber \\&\quad +\Big (\frac{69b^2}{2}-3 \Big )\mathrm {cos}^3(\gamma ) \mathrm {sin}(\gamma )\nonumber \\&\quad + \Big (33b-\frac{15b^3}{2}\Big )\mathrm {cos}^2(\gamma ) \nonumber \\&\quad +\Big (3-\frac{39b^2}{2} \Big )\mathrm {cos}(\gamma ) \mathrm {sin}(\gamma ) +b^3-6b, \end{aligned}$$

230 $$\begin{aligned} b_{124}&:= \Big ( \frac{15c^3}{2}-27c\Big )\mathrm {cos}^4 (\gamma )\nonumber \\&\quad +\Big (\frac{69c^2}{2}-3 \Big )\mathrm {cos}^3(\gamma ) \mathrm {sin}(\gamma )\nonumber \\&\quad + \Big ( 33c-\frac{15c^3}{2}\Big )\mathrm {cos}^2(\gamma ) \nonumber \\&\quad +\Big (3-\frac{39c^2}{2} \Big )\mathrm {cos}(\gamma ) \mathrm {sin}(\gamma ) +c^3-6c, \end{aligned}$$

231 $$\begin{aligned} b_{030}&:= -\frac{\mathrm {sin}^2(\gamma )}{2}[\mathrm {cos}^2(\gamma )-2\mathrm {cos}(\gamma )+1] , \end{aligned}$$

232 $$\begin{aligned} b_{031}&:= \frac{\mathrm {sin}(\gamma )}{6}[15b~\mathrm {cos}^3(\gamma )+23~\mathrm {cos}^2(\gamma )\mathrm {sin}(\gamma )\nonumber \\&\quad -9b~\mathrm {cos}(\gamma )-5\mathrm {sin}(\gamma ) ] , \end{aligned}$$

233 $$\begin{aligned} b_{032}&:= \frac{\mathrm {sin}(\gamma )}{6}[15c~\mathrm {cos}^3(\gamma )+23~\mathrm {cos}^2(\gamma )\mathrm {sin}(\gamma )\nonumber \\&\quad -9c~\mathrm {cos}(\gamma )-5\mathrm {sin}(\gamma ) ] , \end{aligned}$$

234 $$\begin{aligned} b_{033}&:= \Big (\frac{5b^3}{2}-9b \Big )\mathrm {cos}^3(\gamma )\mathrm {sin}(\gamma )\nonumber \\&\quad +\Big (\frac{23b^2}{2}-1\Big )\mathrm {cos}^2(\gamma )\mathrm {sin}^2(\gamma )\nonumber \\&\quad +\Big (9b-\frac{3b^3}{2}\Big )\mathrm {sin}(\gamma )\mathrm {cos}(\gamma )\nonumber \\&\quad +\Big (1-\frac{5b^2}{2} \Big )\mathrm {sin}^2(\gamma ) , \end{aligned}$$

235 $$\begin{aligned} b_{034}&:= \Big (\frac{5c^3}{2}-9c \Big )\mathrm {cos}^3(\gamma )\mathrm {sin}(\gamma )\nonumber \\&\quad +\Big (\frac{23c^2}{2}-1\Big )\mathrm {cos}^2(\gamma )\mathrm {sin}^2(\gamma )\nonumber \\&\quad +\Big (9c-\frac{3c^3}{2}\Big )\mathrm {sin}(\gamma )\mathrm {cos}(\gamma )\nonumber \\&\quad +\Big (1-\frac{5c^2}{2} \Big )\mathrm {sin}^2(\gamma ). \end{aligned}$$

## Appendix B: Stationary solution $\hat{u}_{x}$ and $\hat{v}_{x}$

The stationary solution follows from Eqs. (31) and (32) by considering no time dependence, yielding

236 $$\begin{aligned}&-\frac{E}{M}~ \frac{\partial }{\partial x} \Big ( ~\hat{u}_{x}+\frac{\hat{v}_{x}^2}{2} \Big )=0 \end{aligned}$$

237 $$\begin{aligned}&{ E I_{y} \over A M } \hat{v}_{xxxx} -\frac{T_{0}}{AM}\hat{v}_{xx} -\frac{E}{M} \frac{\partial }{\partial x} \Big [ \hat{v}_{x} \Big ( \hat{u}_{x}+\frac{\hat{v}_{x}^{2}}{2}\Big ) \Big ]\nonumber \\&\quad = (L-x)~g ~\mathrm {sin}(\alpha ) \hat{v}_{xx} -g ~\mathrm {sin}(\alpha ) \hat{v}_{x} \nonumber \\&\qquad +g ~\mathrm {cos}(\alpha ) -{\frac{\rho _{a}}{2AM}} d L v_{\infty }^2 A_{00}. \end{aligned}$$

It will be assumed that $\hat{u}$ is $\mathcal {O}(\epsilon ^2)$, $\hat{v}$ is $\mathcal {O}(\epsilon )$, $\tilde{A}$ is $\mathcal {O}(\epsilon )$, $g \mathrm {sin(\alpha )}=P_{0}^{*}$ is $\mathcal {O}(1)$, $\frac{E}{M}=P_{1}^{*}$ is $\mathcal {O}({1}/{\epsilon })$, ${ E I_{y} \over A M }=P_{2}^{*}$ is $\mathcal {O}({1}/{\epsilon })$, and ${ T_{0} \over A M }=P_{3}^{*}$ is $\mathcal {O}({1}/{\epsilon })$, where $\epsilon$ is a small parameter with $0<\epsilon \ll 1$. Then, by using these assumptions, Eqs. (236) and (237) become

238 $$\begin{aligned}&-P_{1}^{*}~ \frac{\partial }{\partial x} \Big ( ~\hat{u}_{x}+\frac{\hat{v}_{x}^2}{2} \Big )= 0, \end{aligned}$$

239 $$\begin{aligned}&P_{2}^{*} \hat{v}_{xxxx}-P_{3}^{*} \hat{v}_{xx} -P_{1}^{*} \frac{\partial }{\partial x} \Big [ \hat{v}_{x} \Big ( \hat{u}_{x}+\frac{\hat{v}_{x}^{2}}{2}\Big ) \Big ]\nonumber \\&\quad = \frac{\partial }{\partial x} \Big [ P_{0}^{*} (L-x)g \mathrm {sin}(\alpha ) \hat{v}_{x}\Big ]\nonumber \\&\qquad + P_{4}^{*} -{\frac{\rho _{a}}{2AM}} d L v_{\infty }^2 A_{00} +\mathcal {O}(\epsilon ). \end{aligned}$$

with the boundary conditions

240 $$\begin{aligned}&\hat{u}(L)=\hat{u}(0)=0, \nonumber \\&\hat{v}(0)=\hat{v}_{xx}(0)=\hat{v}_{x}(L)=0, \nonumber \\&EI_{y}\hat{v}_{xxx}(L) = T_{0} \hat{v}_{x}(L). \end{aligned}$$

When Eqs. (238) and (239) are integrated with respect to *x*, we may rewrite

241 $$\begin{aligned}&Y\Big ( ~\hat{u}_{x}+\frac{\hat{v}_{x}^2}{2} \Big )= k_{1}, \end{aligned}$$

242 $$\begin{aligned}&\hat{v}_{xxx}-\frac{P_{3}^{*}}{P_{2}^{*} } \hat{v}_{x} -\frac{P_{1}^{*}}{P_{2}^{*} } \frac{\partial }{\partial x} \Big [ \hat{v}_{x} \Big ( \hat{u}_{x}+\frac{\hat{v}_{x}^{2}}{2}\Big ) \Big ]\nonumber \\&\quad =\frac{P_{0}^{*} }{P_{2}^{*}} (L-x)g \mathrm {sin}(\alpha ) \hat{v}_{x} \nonumber \\&\qquad + \frac{P_{4}^{*} x}{P_{2}^{*} } -{\frac{\rho _{a}}{2AMP_{2}^{*} }} d L v_{\infty }^2 A_{00} x +k_{2}, \end{aligned}$$

where $k_{1}$ and $k_{2}$ are constants of integration. Substitute Eq. (241) into Eq. (242), we obtain

243 $$\begin{aligned} \hat{v}_{xxxx}-\hat{v}_{x}(k_{3}-xk_{4})=k_{2}+xk_{5}, \end{aligned}$$

where $k_{4}=\frac{P_{0}^{*}}{P_{2}^{*}}g \mathrm {sin}(\alpha )$, $k_{3}=\frac{(P_{3}^{*}+P_{1}^{*}k_{1})}{P_{2}^{*}}+L k_{4}$, and $k_{5}=\frac{P_{4}^{*}}{P_{2}^{*}}-\frac{\rho _{a}}{2AMP_{2}^{*} } d L v_{\infty }^2 A_{00}$. In order to solve the non-homogeneous PDE Eq. (243), we will apply the method of variation of parameters and obtain

244 $$\begin{aligned}&\hat{v}(x)=\int _{0}^{x} C_{1} A_{i}\Big (\frac{k_{3}-\bar{x}k_{4}}{k_{4}^{2/3}} \Big ) \mathrm {d}\bar{x} \nonumber \\&\quad +\int _{0}^{x} C_{2} B_{i}\Big (\frac{k_{3}-\bar{x}k_{4}}{k_{4}^{2/3}} \Big ) \mathrm {d}\bar{x} \nonumber \\&\quad -\int _{0}^{x} A_{i}\Big (\frac{k_{3}-\bar{x}k_{4}}{k_{4}^{2/3}} \Big ) \Bigg \{ \int _{0}^{\bar{x}} \Big (\frac{k_{2}+sk_{5}}{Wr(s)} \Big ) B_{i}\Big (\frac{k_{3}-s k_{4}}{k_{4}^{2/3}} \Big ) \mathrm {d}s \Bigg \} \mathrm {d}\bar{x} \nonumber \\&\quad +\int _{0}^{x} B_{i}\Big (\frac{k_{3}-\bar{x}k_{4}}{k_{4}^{2/3}} \Big ) \Bigg \{ \int _{0}^{\bar{x}} \Big (\frac{k_{2}+sk_{5}}{Wr(s)} \Big ) A_{i}\Big (\frac{k_{3}-s k_{4}}{k_{4}^{2/3}} \Big ) \mathrm {d}s \Bigg \} \mathrm {d}\bar{x}+C_{3}. \end{aligned}$$

Here $A_{i}$ and $B_{i}$ are Airy functions, and $C_{1}, C_{2}$ and $C_{3}$ are constants of integration, and

245 $$\begin{aligned}&Wr(x)= A_{i}\Big (\frac{k_{3}-{x}k_{4}}{k_{4}^{2/3}} \Big ) \frac{\mathrm {d}}{\mathrm {d}x} \Big [ B_{i}\Big (\frac{k_{3}-{x}k_{4}}{k_{4}^{2/3}} \Big ) \Big ]\nonumber \\&\quad -B_{i}\Big (\frac{k_{3}-{x}k_{4}}{k_{4}^{2/3}} \Big ) \frac{\mathrm {d} }{\mathrm {d} x} \Big [ A_{i}\Big (\frac{k_{3}-{x}k_{4}}{k_{4}^{2/3}} \Big ) \Big ]. \end{aligned}$$

Substituting Eq. (244) into Eq. (241) and integrated with respect to *x*, we obtain

246 $$\begin{aligned}&\hat{u}(x)= k_{1} x -\frac{1}{2}\int _{0}^{x} \Bigg \{ C_{1} A_{i}\Big (\frac{k_{3}-\bar{x}k_{4}}{k_{4}^{2/3}} \Big ) \nonumber \\&\quad +C_{2} B_{i}\Big (\frac{k_{3}-\bar{x}k_{4}}{k_{4}^{2/3}} \Big ) \nonumber \\&\quad -A_{i}\Big (\frac{k_{3}-\bar{x}k_{4}}{k_{4}^{2/3}} \Big ) \Big [ \int _{0}^{\bar{x}} \Big (\frac{k_{2}+sk_{5}}{Wr(s)} \Big ) B_{i}\Big (\frac{k_{3}-s k_{4}}{k_{4}^{2/3}} \Big ) \mathrm {d}s \Big ] \nonumber \\&\quad +B_{i}\Big (\frac{k_{3}-\bar{x}k_{4}}{k_{4}^{2/3}} \Big ) \Big [ \int _{0}^{\bar{x}} \Big (\frac{k_{2}+sk_{5}}{Wr(s)} \Big ) A_{i}\Big (\frac{k_{3}-s k_{4}}{k_{4}^{2/3}} \Big ) \mathrm {d}s \Big ] \Bigg \} ^2 \mathrm {d}\bar{x}\nonumber \\&\quad +C_{4}. \end{aligned}$$

where $C_{4}$ is a constant of integration. By satisfying boundary conditions of Eq. (240) for stationary solution, we obtain

247 $$\begin{aligned} k_{1}&:=\frac{1}{2L} \int _{0}^{L} \hat{v}_{\bar{x}}^2 \mathrm {d}\bar{x}, \end{aligned}$$

248 $$\begin{aligned} k_{2}&:=\frac{N_{3}F_{1}-F_{3}N_{1}}{N_{1}F_{2}+F_{1}N_{2}}, \end{aligned}$$

249 $$\begin{aligned} C_{1}&:=-C_{2}\frac{B_{i} \Big (1, \frac{k_{3}}{k_{4}^{2/3}} \Big )}{A_{i} \Big (1, \frac{k_{3}}{k_{4}^{2/3}} \Big )}, \end{aligned}$$

250 $$\begin{aligned} C_{2}&:=\frac{N_{3}F_{2}+F_{3}N_{2}}{N_{1}F_{2}+F_{1}N_{2}}, \end{aligned}$$

251 $$\begin{aligned} C_{3}&:=C_{4}=0, \end{aligned}$$

where

252 $$\begin{aligned} N_{1}&:= \Bigg \{- A_{i} \Big (\frac{k_{3}-Lk_{4}}{k_{4}^{2/3}} \Big ) \frac{B_{i} \Big (1, \frac{k_{3}}{k_{4}^{2/3}} \Big )}{A_{i} \Big (1, \frac{k_{3}}{k_{4}^{2/3}} \Big )}\nonumber \\&\quad +B_{i} \Big (\frac{k_{3}-Lk_{4}}{k_{4}^{2/3}} \Big ) \Bigg \}, \end{aligned}$$

253 $$\begin{aligned} N_{2}&:=\Bigg \{ -A_{i} \Big (\frac{k_{3}-Lk_{4}}{k_{4}^{2/3}} \Big ) \int _{0}^{L} \frac{1}{Wr(\bar{x})}B_{i} \Big (\frac{k_{3}-\bar{x}k_{4}}{k_{4}^{2/3}} \Big ) \mathrm {d}\bar{x}\nonumber \\&\quad +{B_{i} \Big (\frac{k_{3}-Lk_{4}}{k_{4}^{2/3}} \Big )}\int _{0}^{L} \frac{1}{Wr(\bar{x})}A_{i} \Big (\frac{k_{3}-\bar{x}k_{4}}{k_{4}^{2/3}} \Big ) \mathrm {d}\bar{x}\Bigg \}, \end{aligned}$$

254 $$\begin{aligned} N_{3}&:=\Bigg \{ A_{i} \Big (\frac{k_{3}-Lk_{4}}{k_{4}^{2/3}} \Big ) \int _{0}^{L} \frac{\bar{x}k_{5}}{Wr(\bar{x})}B_{i} \Big (\frac{k_{3}-\bar{x}k_{4}}{k_{4}^{2/3}} \Big ) \mathrm {d}\bar{x}\nonumber \\&\quad -{B_{i} \Big (\frac{k_{3}-Lk_{4}}{k_{4}^{2/3}} \Big )}\int _{0}^{L} \frac{\bar{x}k_{5}}{Wr(\bar{x})}A_{i} \Big (\frac{k_{3}-\bar{x}k_{4}}{k_{4}^{2/3}} \Big ) \mathrm {d}\bar{x}\Bigg \}, \end{aligned}$$

255 $$\begin{aligned}&F_{1}:=\frac{E I_{y}}{T_{0}}(k_{3}-Lk_{4}) \Bigg \{ B_{i} \Big (\frac{k_{3}-Lk_{4}}{k_{4}^{2/3}} \Big )\nonumber \\&\quad - A_{i} \Big (\frac{k_{3}-Lk_{4}}{k_{4}^{2/3}} \Big )\frac{B_{i} \Big (1, \frac{k_{3}}{k_{4}^{2/3}} \Big )}{A_{i} \Big (1, \frac{k_{3}}{k_{4}^{2/3}} \Big )}\Bigg \}, \end{aligned}$$

256 $$\begin{aligned}&F_{2}:=\frac{E I_{y}}{T_{0}}(k_{3}-Lk_{4}) \Bigg \{ A_{i} \Big ( \frac{k_{3}-Lk_{4}}{k_{4}^{2/3}} \Big ) \int _{0}^{L} \frac{1}{Wr(\bar{x})}B_{i} \Big (\frac{k_{3}-\bar{x}k_{4}}{k_{4}^{2/3}} \Big ) \mathrm {d}\bar{x}\nonumber \\&\quad -{B_{i} \Big ( \frac{k_{3}-Lk_{4}}{k_{4}^{2/3}} \Big )}\int _{0}^{L} \frac{1}{Wr(\bar{x})}A_{i} \Big (\frac{k_{3}-\bar{x}k_{4}}{k_{4}^{2/3}} \Big ) \mathrm {d}\bar{x} \Bigg \}\nonumber \\&\quad +\frac{E I_{y}}{T_{0}} k_{4}^{1/3} \Bigg \{A_{i} \Big (1, \frac{k_{3}-Lk_{4}}{k_{4}^{2/3}} \Big ) \frac{1}{Wr(L)}B_{i} \Big (\frac{k_{3}-Lk_{4}}{k_{4}^{2/3}} \Big ) \nonumber \\&\quad -{B_{i} \Big (1, \frac{k_{3}-Lk_{4}}{k_{4}^{2/3}} \Big )}\frac{1}{Wr(L)}A_{i} \Big (\frac{k_{3}-Lk_{4}}{k_{4}^{2/3}} \Big ) \Bigg \}, \end{aligned}$$

257 $$\begin{aligned}&F_{3}:=\frac{E I_{y}}{T_{0}}(k_{3}-Lk_{4}) \Bigg \{ A_{i} \Big ( \frac{k_{3}-Lk_{4}}{k_{4}^{2/3}} \Big ) \int _{0}^{L} \frac{\bar{x}k_{5}}{Wr(\bar{x})}B_{i} \Big (\frac{k_{3}-\bar{x}k_{4}}{k_{4}^{2/3}} \Big ) \mathrm {d}\bar{x}\nonumber \\&\quad -{B_{i} \Big ( \frac{k_{3}-Lk_{4}}{k_{4}^{2/3}} \Big )}\int _{0}^{L} \frac{\bar{x}k_{5}}{Wr(\bar{x})}A_{i} \Big (\frac{k_{3}-\bar{x}k_{4}}{k_{4}^{2/3}} \Big ) \mathrm {d}\bar{x} \Bigg \} \nonumber \\&\quad +\frac{E I_{y}}{T_{0}} k_{4}^{1/3} \Bigg \{-A_{i} \Big (1, \frac{k_{3}-Lk_{4}}{k_{4}^{2/3}} \Big ) \frac{Lk_{5}}{Wr(L)}B_{i} \Big (\frac{k_{3}-Lk_{4}}{k_{4}^{2/3}} \Big ) \nonumber \\&\quad +{B_{i} \Big (1, \frac{k_{3}-Lk_{4}}{k_{4}^{2/3}} \Big )}\frac{Lk_{5}}{Wr(L)}A_{i} \Big (\frac{k_{3}-Lk_{4}}{k_{4}^{2/3}} \Big ) \Bigg \}. \end{aligned}$$
